# Comparison of the complete genome sequence of two closely related isolates of ‘*Candidatus* Phytoplasma australiense’ reveals genome plasticity

**DOI:** 10.1186/1471-2164-14-529

**Published:** 2013-08-02

**Authors:** Mark T Andersen, Lia W Liefting, Ilkka Havukkala, Ross E Beever

**Affiliations:** 1 The New Zealand Institute for Plant & Food Research Limited, Private Bag 92169, Auckland 1142, New Zealand; 2 AgriGenesis Biosciences Ltd, P.O. Box 50, Auckland, New Zealand; 3 Current address: Plant Health and Environment Laboratory, Ministry for Primary Industries, P.O. Box 2095, Auckland 1140, New Zealand; 4 Current address: Intellectual Property Office of New Zealand, 205 Victoria Street, Wellington, New Zealand; 5 Landcare Research, Private Bag 92170, Auckland 1142, New Zealand

**Keywords:** Phytoplasma, Genome sequence, Synteny, Rearrangement, Plasticity, Potential mobile unit (PMU)

## Abstract

**Background:**

‘*Candidatus* Phytoplasma australiense’ is associated with at least nine diseases in Australia and New Zealand. The impact of this phytoplasma is considerable, both economically and environmentally. The genome of a NZ isolate was sequenced in an effort to understand its pathogenicity and ecology. Comparison with a closely related Australian isolate enabled us to examine mechanisms of genomic rearrangement.

**Results:**

The complete genome sequence of a strawberry lethal yellows (SLY) isolate of ‘*Candidatus* Phytoplasma australiense’ was determined. It is a circular genome of 959,779 base pairs with 1126 predicted open reading frames. Despite being 80 kbp larger than another ‘*Ca*. Phytoplasma australiense’ isolate PAa, the variation between housekeeping genes was generally less than 1% at a nucleotide level. The difference in size between the two isolates was largely due to the number and size of potential mobile units (PMUs), which contributed to some changes in gene order. Comparison of the genomes of the two isolates revealed that the highly conserved 5′ UTR of a putative DNA-directed RNA polymerase seems to be associated with insertion and rearrangement events. Two types of PMUs have been identified on the basis of the order of three to four conserved genes, with both PMUs appearing to have been present in the last common ancestor of ‘*Ca*. Phytoplasma asteris’ and ‘*Ca*. Phytoplasma australiense’. Comparison with other phytoplasma genomes showed that modification methylases were, in general, species-specific. A putative methylase (*xor*IIM) found in ‘*Ca*. Phytoplasma australiense’ appeared to have no analogue in any other firmicute, and we believe has been introduced by way of lateral gene transfer. A putative retrostransposon (*ltrA*) analogous to that found in OY-M was present in both isolates, although all examples in PAa appear to be fragments. Comparative analysis identified highly conserved 5′ and 3′ UTR regions of *ltrA*, which may indicate how the gene is excised and inserted.

**Conclusions:**

Comparison of two assembled ‘*Ca*. Phytoplasma australiense’ genomes has shown they possess a high level of plasticity. This comparative analysis has yielded clues as to how rearrangements occur, and the identification of sets of genes that appear to be associated with these events.

## Background

Phytoplasmas are pathogenic bacteria associated with diseases of several hundred plant species, with many newly emerging diseases being reported [1]. Symptoms typical of phytoplasma infection are virescence (green flowers), phyllody (development of leaf structures instead of flowers), proliferation, stunting and yellowing – symptoms that mimic hormone imbalances and/or perturbations of normal plant development. Phytoplasmas belong to the Mollicutes, the class of bacteria characterised by an absence of cell walls, and small (0.530 – 1.350 Mbp) genomes as a result of genome reduction [2]. Despite considerable efforts, a procedure to culture phytoplasmas axenically has yet to be developed, which has hindered research into these economically important organisms.

Apart from Poinsettia-branch-inducing phytoplasma found in poinsettia (*Euphorbia pulcherrima*), the only other phytoplasmas found in New Zealand belong to ‘*Candidatus* Phytoplasma australiense’ [3-5]. *Phormium* yellow leaf (PYL) was first reported in 1908, but was only determined to be a phytoplasma-associated disease in 1969 [6]. Since the late 1970s, this phytoplasma has been discovered associated with diseases in New Zealand of *Cordyline*, *Coprosma*, strawberry, and more recently potato [7-11]. In addition, ‘*Ca*. Phytoplasma australiense’ is associated with at least five diseases in Australia [12-14]. Although the 16S rRNA gene sequences of all isolates are very similar, sequence variation observed in protein coding genes, such as *tuf* and *rp*, has enabled sub-species differentiation [15-17]. There has been nothing, however, about the groupings that could explain the emergence of the more recent diseases, as representatives of the two *tuf* groupings found in New Zealand were associated with both PYL and the more recently emerged diseases. That ‘*Ca*. Phytoplasma australiense’ is being detected in increasing numbers of plant species has heightened the need to devise effective methods to control or manage the pathogen.

The development of molecular tools has greatly assisted in the detection, identification and classification of phytoplasmas. Further advances in understanding the biology of phytoplasmas and how they cause disease are being made with the sequencing and analysis of complete genomes.

To date, the genome sequences of four phytoplasmas have been published. They are onion yellows mild strain (OY-M) [18], aster yellows witches’-broom (AY-WB) [19], ‘*Ca.* Phytoplasma australiense’ Australian isolate (PAa) [14], and apple proliferation phytoplasma (AT) [20]. Analysis of these genomes has revealed that phytoplasmas lack numerous biochemical pathways previously thought to be essential for a living organism. In particular, phytoplasmas lack the ability to synthesise many fundamental biochemicals essential to bacteria [21], and instead rely on their importation from the environment. This is reflected in the numerous transporters contained in their genomes. As many as 27 transporter genes were reported in OY-M [18] and more than 30 in PAa [14]. Despite the propensity for phytoplasmas to undergo genome reduction, most phytoplasma genomes consist of repeated elements that consist of genes associated with gene replication and transposition, termed Potential Mobile Units (PMUs) [19]. Concern about the true nature of inverse repeats at the boundaries of PMUs causes some researchers to prefer the term sequence-variable mosaics, as they believe that it more accurately reflects the origin and function of these units [22]. These authors contend that rather than being mobile units, the areas are hot-spots for insertion by mobile elements, and their composition is a result of multiple targeted bacteriophage attacks [23,24]. It has been hypothesised that such areas could be “gene factories” where new genes are created through rearrangement, insertion, deletion and gene fusion. Furthermore, it has been proposed that the genes associated with PMUs –referred to as mobile unit genes (MUGs) - are distinct from genes positioned elsewhere in the genome – termed fundamental genes or FUGs [25].

Whole genome analyses have revealed the considerable diversity in the genus *Candidatus* Phytoplasma. AT, which at 602 kbp is the smallest of the published phytoplasma genomes, differs significantly from the others. Unlike the other published genomes, it is composed of a linear chromosome with repeated arrangements at the arm ends, and a conserved core of housekeeping genes. Furthermore, AT does not appear to have PMUs other than isolated gene remnants, but does, however, have a greater complement of genes associated with homologous recombination and excision repair [20].

Phytoplasma genomics has also led to the identification of genes that could be involved with pathogenicity and virulence. Such genes are those annotated as haemolysins and adhesion-related proteins, as well as genes associated with insect transmission [26]. Duplication of a set of glycolytic genes in the virulent strain of onion yellows has been proposed as a cause of increased pathogenicity [27]. More recently, studies of potential pathogenicity factors have focused on proteins with putative signal peptides, identifying proteins capable of nuclear localisation [28], as well as phytoplasma genes that produce phytoplasma-like symptoms when inserted into *Arabidopsis* plants [29].

The complete genome of a SLY isolate (NZSb11) of ‘*Ca*. Phytoplasma australiense’ is presented, together with a comparison with PAa, another isolate from the same species. This comparison enabled us to examine the variation that occurred over a comparatively short phylogenetic difference and to contribute further to understanding the genetic requirements and evolution of these enigmatic plant pathogens.

## Results

### General features of the ‘*Ca.* Phytoplasma australiense’ SLY isolate genome

**Figure 1:**
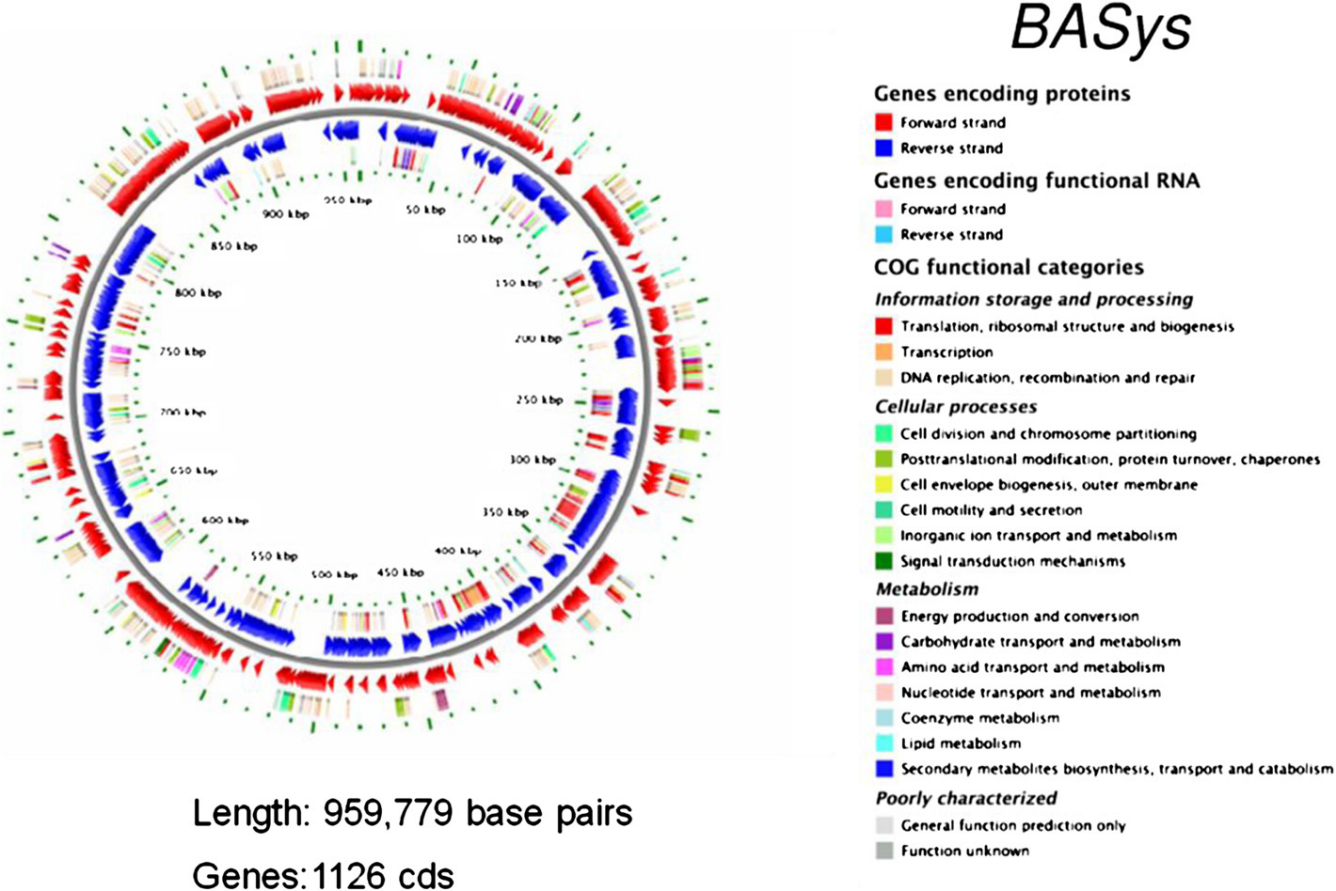
**Circular representation of the chromosome of SLY isolate of ‘*Candidatus *Phytoplasma australiense’.** The genome sequence was annotated by BASys (Bacterial Annotation System). The protein coding genes on the forward (red arrows) and reverse (blue arrows) strands are shown. The Clusters of Orthologous Groups (COG) functional categories of the protein coding genes in the forward (outermost circle) and reverse (innermost circle) directions are colour-coded as designated in the inset. Allocation to a particular COG functional group was determined by BLAST searches against protein databases as described in van Domselaar *et al.*[44].

The genome of ‘*Ca*. Phytoplasma australiense’ NZSb11 (hereafter referred to as SLY) consists of a single circular chromosome of 959,779 bp (Figure 1) and a single plasmid of 3,635 bp (pPASb11). Sequence analysis of pPASb11 was reported previously [10]. In accordance with the precedent established with OY-M, the initiation codon for the *dna*A gene was chosen as the start point for numbering the genome. There are 1126 ORFs in SLY genome that are predicted to be protein-coding genes (see Additional file 1), two rRNA operons, and 35 tRNAs (one of which is a pseudo tRNA). The majority of translation initiation codons for the predicted protein-coding genes are ATG (777), while GTG (73) and TTG (276) are also proposed. A putative function has been assigned to 528 ORFs, while 598 ORFs have no assigned function, and are described as hypothetical proteins. In accordance with previous phytoplasma genome sequencing projects, TGA was interpreted as a stop codon. The general features and comparison with other phytoplasma genomes is summarised in Table 1.

**Table 1:** Comparison of phytoplasma genomes

Strain	**SLY**	**PAa**	**OY-M**	**AYWB**	**AT**
Length (bp)	959,779	879,324	860,631	706,569	601,943
G + C content (%)	27	27	28	27	21.4
Protein-coding region (%)	78	74	73	72	78.9
No. protein-coding genes with assigned function	528	502	446	450	338
No. of conserved hypothetical genes	249	214	51	149	72
No. of hypothetical genes	349	123	257	72	87
Total no. genes	1126	839	754	671	497
No. of tRNA genes	35	35	32	31	32
No. of rRNA operons	2	2	2	2	2

General features of the chromosomes of complete phytoplasma genomes for ‘*Candidatus* Phytoplasma australiense’ isolates SLY and PAa, ‘*Candidatus* Phytoplasma asteris’ isolates OY-M and AYWB, and ‘*Candidatus* Phytoplasma mali’ isolate AT.

Gene duplications and repeated areas are prevalent in the SLY genome. The most notable is a region of approximately 12612 bp between positions 134234–146846, which is 100% identical at a nucleotide level to the region 497732–510344. These represent ORFs between SLY160-180 and SLY571-591. At one end, the repeat sequence ends abruptly, corresponding to c. 140 bp of the 5′ UTR of *rpoD*. Other areas of direct repeats are between SLY185 and SLY190, which corresponds to the area between SLY595 and SLY600, and an area between SLY617 and SLY622, which is duplicated twice more between SLY704 and SLY699, and SLY970 and SLY965.

Of 1126 ORFs predicted for the SLY genome, approximately 600 are present in the genome as a single copy. There are only 47 putative genes (annotated and HPs) that are present in more than one copy and 26 that occur more than 6 times (including probable pseudo genes). Of putative genes with more than 6 copies, all but one (*ltrA*) feature in clusters that are duplicated numerous times throughout the genome. These 229 clusters are similar in gene composition and structure to the genomic features previously described as potential mobile units (PMUs).

In SLY we identified 14 clusters of repeated genes that corresponded in composition to the PMUs described by Bai *et al.*[19]. The margins of each PMU area are listed in Table 2. In most cases the boundaries of the PMU were represented by a *tra5* transposase, although other determinants were *rpoD*, putative prophage protein *gepA*, ATP-dependent Zn protease *hflB*, and CHPs (Conserved Hypothetical Proteins) closely associated with the repeated gene clusters. Although referred to as PMUs, the regions described may contain more than one “unit” and may reflect a coalescence of numerous “PMUs”. The sequences of the PMUs as defined constitute 378950 base pairs, which amount to 39% of the SLY genome. See Additional file 2 for graphic depiction of their distribution in the genome.

**Table 2:** Potential mobile units in SLY

**PMU#**		**ORFs**			**Bases**		**Size (bp)**
	**Start**		**Finish**		**Start**	**Finish**	
1	SLY033	*tra5*	SLY071	CHP	34885	63505	28620
2	SLY107	*tra5*	SLY136	*tra5*	94133	113691	19558
3	SLY147	*tra5*	SLY192	*tra5*	123952	159925	35973
4	SLY203	*tra5*	SLY230	*tra5*	171531	192821	21290
5	SLY247	CHP	SLY261	*tra5*	208477	216439	7962
6	SLY458	*hflB*	SLY467	*tra5*	389597	396448	6851
7	SLY554	*tra5*	SLY646	CHP	481516	555345	73829
8	SLY683	*rpoD*	SLY734	*rpoD*	588005	624217	36212
9	SLY763	*tra5*	SLY787	*rpoD*	653052	674028	20976
10	SLY811	*tra5*	SLY835	HP	696660	715858	19198
11	SLY849	*tra5*	SLY858	*rpoD*	728312	736162	7850
12	SLY922	*tra5*	SLY1006	*tra5*	798788	864178	65390
13	SLY1020	*rpoD*	SLY1043	*tra5*	877646	890041	12395
14	SLY1073	*rpoD*	SLY1105	*tra5*	914765	937611	22846

Size of SLY Potential Mobile Units (PMU) areas as determined using Open Reading Frame (ORF) boundaries. Each area consists of genes associated with PMUs, and may consist of one or more “units”.

### Comparison between PAa and SLY genome sequences

A comparison of putative ORFs in the 61% of the genome that is not composed of PMUs indicates that the gene complements of the two ‘*Ca*. Phytoplasma australiense’ isolates PAa and SLY are almost identical. Of the annotated ORFs, there are only two that are present in SLY but absent in PAa. They are three fragments of a putative *recA* gene (SLY1046-1048), and SLY017 (*yecS* – “Inner membrane amino-acid ABC transporter permease protein”). In PAa, there are a number of annotated ORFs that are not present in SLY. PA0438, for example, is annotated as a restriction-modification enzyme “restriction enzyme alpha subunit”. Other examples are ORFs associated with the two “PMU5” found in PAa, which in general are not found in SLY. One exception is SLY504, annotated as *dnaB*, which at the amino acid level has the greatest similarity (83%) with PA0361 - a DNA helicase associated with PAa-PMU5.

At 959779 bp, the SLY genome is 80455 bp larger than PAa. The difference in the size is largely due to the number and size of PMUs in the SLY genome. In PAa, the 12 PMUs appear to constitute a lower percentage of the genome than the 39% assessed for SLY. Although Tran-Nguyen et al. [14] cites 106,682 bp or 12.1% of the PAa genome as being in PMU clusters, their definition of what constituted a PMU was more conservative than ours. Using parameters similar to those for SLY, we estimate the comparable figure for PAa is 310588 bp, which constitutes 35% of the genome (see Additional file 3).

The DNA sequence of 23 single copy genes - not associated with PMUs - from SLY and PAa were compared. The similarity at a nucleic acid level was never lower than 99%, even in *malE* which contained a 15 bp insertion-deletion (INDEL). In contrast, a subset of 19 of these genes from the two isolates from ‘*Ca*. Phytoplasma asteris’ (OY-M and AY-WB) ranged from 91% to 97% (see Additional file 4).

**Figure 2:**
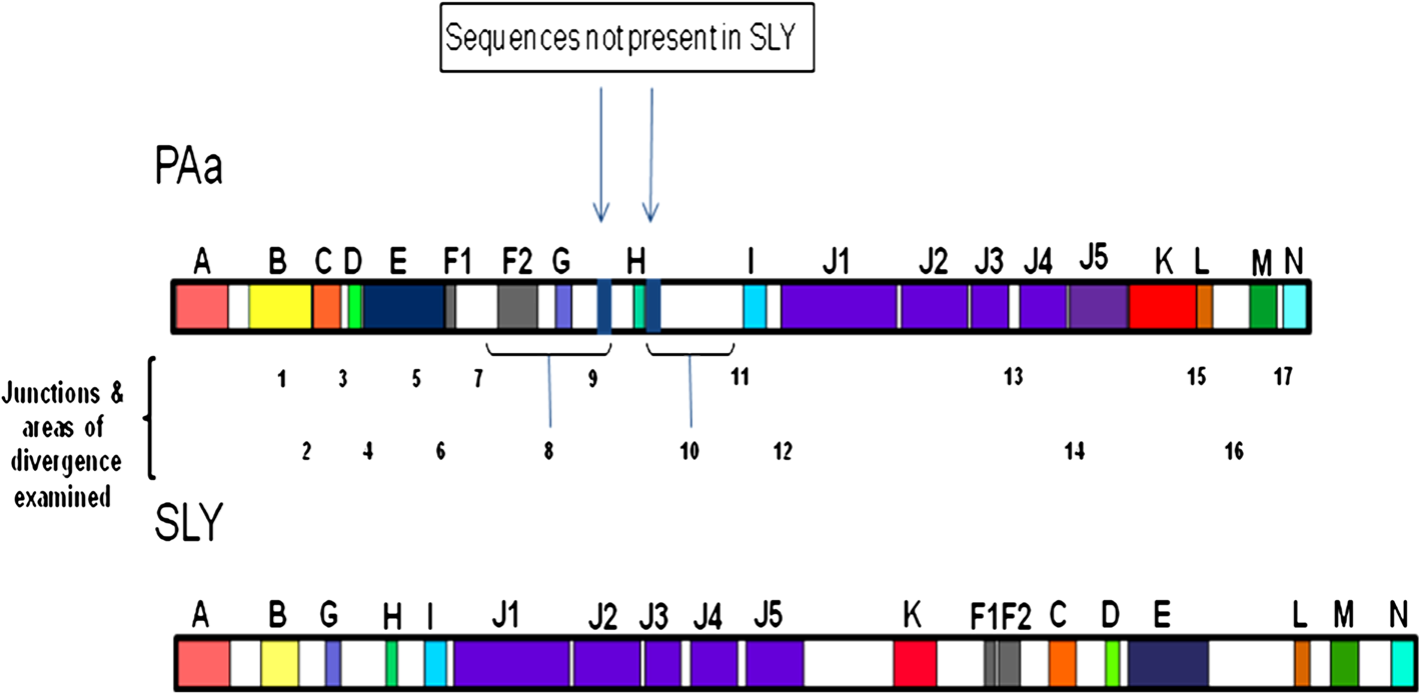
**Graphic representation of linearised genome sequences of ‘*Candidatus *Phytoplasma australiense’ isolates PAa and SLY.** Coloured blocks represent unambiguous orthologous Open Reading Frames (ORFs) using PAa as the reference strain. Seventeen junctions where the two sequences diverged either by insertions, deletions or rearrangements are labelled below the representation of the PAa genome.

Although the housekeeping genes were >99% similar at a nucleotide level, gene order differed between the two isolates. A comparison of ORFs not associated with PMUs revealed syntenic variation between SLY and PAa (Figure 2 and Table 3). The blocks have been labelled “A” to “N”, with PAa used as the reference strain. The most obvious differences were that in SLY the blocks labelled “C” to “E” were located between blocks “F” and “L”. Block “F” in SLY appeared after block “K” and was a single unit, but in PAa this cluster was divided into “F1” and “F2” as a result of a 30 kbp INDEL (PMU insertion). Block “J” was divided into 5, as there were several areas of differences within an otherwise ostensibly syntenous region. We note that two examples of the “PAa-PMU5” were situated either side of block “H” in PAa, whereas this type of PMU was not found in SLY.

**Table 3:** **Syntenous blocks of ‘*Ca*. Phytoplasma australiense’ isolates PAa and SLY**

**Label**	**SLY**	**SLY**	**SLY**	**SLY**	**PAa**	**PAa**
**(PAa)**	**ORF**	**ORF**	**Nucleotide**	**Nucleotide**	**Nucleotide**	**Nucleotide**
	**Start**	**Finish**	**Start**	**Finish**	**Start**	**Finish**
A	001	040	1	40265	1	40101
B	072	106	64426	93481	56483	105587
C	788	810	674805	696078	106479	127765
D	839	850	719059	729267	133720	143959
E	859	921	736461	798394	145877	208544
F1	735	745	624885	633062	209078	217257
F2	747	762	635349	652744	250091	281359
G	138	150	114224	126090	295457	307489
H	194	200	161365	169875	356035	364551
I	231	246	190938	208168	441065	459039
J1	257	349	214297	303031	470769	560305
J2	354	428	306791	358539	564063	615798
J3	433	458	362082	389833	618865	647498
J4	468	501	397061	433724	656566	693092
J5	509	557	439943	484888	695292	740409
K	645	682	554567	587867	741206	793799
L	1007	1019	864691	877435	793878	806622
M	1051	1072	892855	914649	835315	856746
N	1106	1126	939870	959149	861075	879438

Syntenous blocks of ‘*Candidatus* Phytoplasma australiense’ isolates PAa and SLY, as determined by unambiguous orthologus Open Reading Frames (ORFs). Nucleotide starts and finishes correspond to the starts and finishes of the ORFs and are not intended to indicate accurately and precisely where the junctions of orthologous sequence begin and end.

Seventeen areas where the sequence of the two isolates diverged were examined (Figure 2). In three cases areas 1, 2 and 6, junctions were marked by sequence overlaps ranging from four to eleven base pairs. No pattern was evident, however, as none was the same, and none corresponded to an obvious restriction endonuclease site, which might indicate a mechanism of insertion or excision. In areas 8 and 14 where the genomes differed as a result of an insertion event, direct repeats were identified at both ends of the insertion. These were either 4 bp or 39 bp, with the larger associated with a 4 kbp insert in between blocks “J4” & “J5” of SLY. These direct repeats could be examples of footprints left as a result of a duplication / insertion event.

The type of junction at area 6 was the most prevalent type of junction observed. This variation in sequence occurred at the end of Block “E”, and signalled a major difference in synteny. In PAa, Block “E” led into Block “F”, whereas in SLY it led into a PMU prior to Block “L”. When SLY sequence was superimposed on PAa, there was a four-base overlap of “GAAT” 41 bp 5′ of SLY922 (orientated counter-clockwise). More significantly, perhaps, this junction corresponded to 140 bp of the 5′ UTR region of SLY734 (*rpoD*) in the SLY genome.

### PMUs of ‘*Ca*. Phytoplasma australiense’

Of the more than 30 distinct ORFs found within the areas designated as PMUs, there are a number that are highly conserved. Amongst these are a group of co-located three genes: thymidylate kinase (*tmk*), a conserved hypothetical protein that appears to be associated with *tmk*, and a DNA helicase.

There are 21 ORFs that are annotated as thymidylate kinase (*tmk*) in the SLY genome, and these can be further classified into *tmk-a* and *tmk-b*, as proposed for OY phytoplasma [30]. In SLY there is one copy of *tmk-b* (SLY918) and 20 copies of *tmk-a*, although one (SLY466) is truncated and should be considered a pseudo-gene. The position of *tmk-b* in all published phytoplasma genomes is conserved, and is not associated with a PMU.

In SLY all *tmk-a* ORFs follow a conserved hypothetical protein, which we have described as “CHPtap” (*tmk*-associated conserved hypothetical protein). The one exception is in SLY-PMU5, where the *tmk-a* is severely truncated because of a gross deletion after 33 nucleotides (truncated to the extent that it does not register as an ORF). There are 21 copies of CHPtap in the SLY genome, and they are very highly conserved. CHPtap appears to be highly conserved throughout the genus, with analogues detected in OY-M, AY-WB, PAa, clover phyllody (ABA25862), Loofah witches’ broom (AAK54674), and ‘*Ca*. Phytoplasma solani’ (CAJ17869 and CAJ17791). CHPtap is 60% - 70% similar with the analogues in these phytoplasmas, except for PAa where the similarities are greater than 90%. No discernable analogue was detected, however, in apple proliferation phytoplasma. In SLY, the ORFs of CHPtap and *tmk-a* overlap by 11 nucleotides, suggesting that this gene arrangement could be operonal. Both *tmk-a* and CHPtap are closely associated with a DNA helicase (*dnaC* or *B*), although 8 of 30 putative *dnaC*/*B* genes are not associated with CHPtap and *tmk-a*.

In SLY there are 26 ORFs that are annotated as *dnaC*. Of these, only two (SLY106 and SLY888) are not associated with a PMU. The putative full-length *dnaC* gene products associated with PMUs have 79% identity when compared with one another, but with only 30% and 50% identity to SLY106 and SLY888 respectively. When compared with *dnaC* from other phytoplasmas, SLY888 was identical (100%) to PA0148, with 72% identity to PAM013 and AYWB_007, which were annotated as DNA helicase “*dnaB*”, and 50% with ATP_00176, annotated as *dnaB1*. Furthermore, the position of this ORF was highly conserved in the genome, situated in all cases upstream of peptide chain release factor (*prfA*), and downstream of 50S ribosomal protein L9 (*rplI*). SLY106 is 96% similar to PA0094, and the two are syntenic, although the synteny of the two genomes differs after the ORF. No obvious orthologue was detected in other phytoplasmas, and there was no more than 30% identity with other *dnaC* annotated proteins. It has been annotated as a “replicative helicase” because of strong similarity at the carboxyl terminus, and it has greatest similarity with the *dnaC* associated with PMUs. The putative protein sequences of the *dnaC* genes associated with PMUs from SLY had 51% - 56% identity to that of PAM428, compared with 21% - 26% with the other PMU-associated DNA helicases in OY-M.

SLY1064 is annotated as *dnaB*, and is orthologous to PA0822 (100%), PAM673 (48%), AYWB_068 (49%) and AP_00070 (50%), with identities in brackets. SLY504, also annotated as *dnaB*, is orthologous to a number of ORFs in PAa (78%), AY-WB and OY-M, all of which seem to be associated with PMUs. OY-M and AY-WB have only one ORF annotated as *dnaC* (PAM627 and AYWB_069). The proteins from those putative genes had 52% identity to SLY1063, which is annotated as *dnaI* “primosome subunit”. In PAa the orthologue is PA0821 (100%), which is annotated as “tmRNA” – “putative DNA replication protein”.

**Figure 3:**
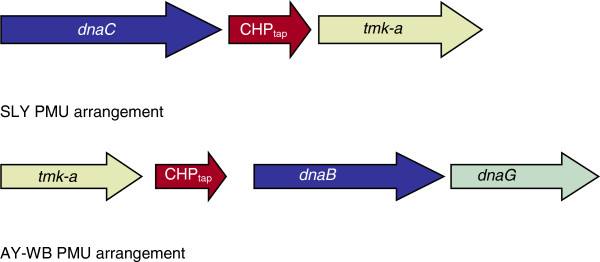
**Conserved core genes of PMUs.** Gene order (5′-3′) of three and four highly conserved genes found in the Potential Mobile Units (PMUs) of ‘*Ca*. Phytoplasma australiense’ and ‘*Ca.* Phytoplasma asteris’. The “AY-WB PMU” arrangement is the only one found in AY-WB, the “SLY PMU” arrangement is the only type found in SLY, whereas OY-M and PAa have examples of both types.

There appear to be two distinct types of PMU based on the arrangement of three to four key ORFs. These ORFs are annotated as *tmk-a*, *dnaC* or *B*, *dnaG*, and a conserved hypothetical protein (CHPtap) that appears to be associated with *tmk-a*. The arrangement of these ORFs in AY-WB PMUs is in the order (5′-3′) *tmk-a*, CHPtap, *dnaB*, and *dnaG*. In SLY these ORFs are (5′-3′) *dnaC*, CHPtap, and *tmk-a*, with no *dnaG* (Figure 3). In PAa, the majority of PMUs have the arrangement as found solely in SLY; however, two of the AY-WB arrangements are also present. In OY-M, only one example of the SLY arrangement can be detected, and that is in the PMU containing ORFs 428–430. Although the DNA helicase (PAM428) is of comparable size to the DNA helicases from PMUs of SLY, PAM429 and PAM430 are truncations of the respective ORFs found in the SLY PMUs. Consequently we conclude that this OY-M PMU seems to be accumulating errors and is possibly in the process of being eliminated.

There are a number of other putative genes that appear to be conserved in the PMUs. Two classes that seem to be associated with differences between PAa and SLY are those annotated as methylases, and those annotated as sigma factors associated with DNA-directed RNA polymerases.

**Figure 4:**
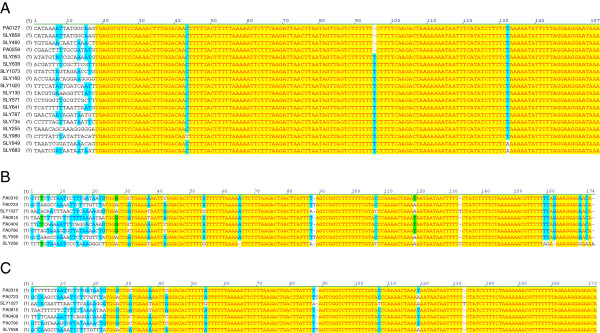
**5**′ **UTR of *rpoD*.** DNA sequence alignments of the 5′ Untranslated Region (UTR) of **(A)***rpoD* Group A and **(B)***rpoD* Group B genes found in ‘*Ca.* Phytoplasma australiense’. **(C)** Alignment of 5′ UTR region of Group B without SLY256. Although SLY256 has the best match with Group B, its protein sequence indicates that it is the most divergent of examples within that group. There is greater conservation of the 5′ UTR regions of the remaining members of Group B, although they seem to be more divergent that those of Group A. Yellow colouring indicates exact nucleotide match and blue colouring is greatest consensus match.

There are 20 ORFs in the SLY genome that are annotated as *rpoD* - DNA-dependent RNA polymerase. Those ORFs annotated as *rpoD* are all associated with PMUs and are quite distinct from the DNA-dependent RNA polymerase not associated with a PMU (SLY419), which is annotated as *sigA*. In PAa there are five ORFs annotated as *fliA* (DNA-directed RNA polymerase, specialized sigma subunit). They are PA0316, PA0409, PA0723, PA0790 and PA0815. There are also two ORFs (PA0127 and PA0258) annotated as “RNA polymerase sigma factor”. In SLY the ORFs characterised as DNA-dependent RNA polymerase can be divided into four groups: *sigA*, *rpoA*, *rpoB*, *rpoC*, and *rpoD*. The ORFs annotated as *rpoD* can be further divided into Group A and Group B. The *rpoD A* &*B* groupings are complex because the protein sequences appear to be a mix of motifs that may be shared between otherwise distant sequences and not present in those seemingly more closely related. The groupings are more distinct when the 5′ UTR regions are compared, as the regions of c. 140 bp (GpA) and c. 150 bp (GpB) are highly conserved (Figure 4). Our assessment is that PAa ORFs annotated as *fliA* are orthologous to *rpoD* Group B, whereas PA0127 and PA0258 are orthologous to *rpoD* Group A (see Additional file 5).

The conserved 5′ UTR sequences of *rpoD* appear at the boundaries of a number of insertion / duplication events. The boundary at one end of the exact duplications at position 134234 & 497732 in SLY corresponds to the 140 bp 5′ UTR of *rpoD* GpA. Furthermore at this boundary, SLY570 appears to be disrupted by the intrusion of the 5′ UTR of SLY571. Another example of a gene having been altered by the intrusion of the 5′ UTR of *rpoD* is SLY1026, annotated as methylase llaDCHIA. There is 92% - 100% similarity at the nucleotide level over the 11 putative full-length llaDCHIA ORFs. SLY1026 differs significantly c. 33 nucleotides from the 3′ end of the gene. This sequence corresponds to the conserved 150 bp 5′ UTR of SLY1027 (*rpoD* GpB) (see Additional file 6). The 5′ UTR of *rpoD* is also implicated in the differences between PAa and SLY. Comparison of the syntenic differences between the two ‘*Ca*. Phytoplasma australiense’ isolates revealed that on at least seven occasions the junction where the gene order of the two genomes varied was at the conserved 5′ UTR of *rpoD* (see Additional file 7).

There are five putative modification methylases annotated in the SLY genome, representing 42 ORFs (including fragments). They are *yhhF*, llaDCHIA, *hpa*IM, CHPmethylase and *xor*IIM. SLY507 is annotated as *hpa*IM, but the predicted protein sequence spans a number of methylases compared with the ORFs from PAa (see Additional file 8). Apart from *yhhF* (PAM299), which appears to have orthologues in all phytoplasma genomes, the other methylases tend to be specific to the Candidatus species (Table 4).

**Table 4:** Putative methylases of phytoplasmas

**Methylase**	**OY-M**	**AY-WB**	**AT**	**PAa**	**SLY**
llaDCHIA	-	-	-	1	11 (2)
*hpa*IM	-	-	-	2 (1)^1^	2^2^
CHP_methylase_	-	-	-	12 (1)	13 (3)
*xor*IIM	-	-	-	(2)	5 (5)
Restriction-modification enzyme ‘restriction enzyme alpha subunit’.	-	-	1	1	-
Type II DNA modification methyltransferase	-	-	-	2 (5)	(1)^2^
*dam*	2	1	-	-	-
N-6 DNA methylase	-	-	3	-	-
Type I restriction-modification system methyltransferase subunit	-	-	1	-	-

^1^Fragment is PA0654 that precedes PA0655 and has 87% similarity over the common area.

^2^Includes SLY507, which seems to be a protein that spans regions of a number of methylases.

List of putative methylases reported in ‘*Candidatus* Phytoplasma asteris’ (OY-M and AY-WB), ‘*Ca*. Phytoplasma australiense’ (PAa and SLY), and ‘*Ca*. Phytoplasma mali’ (AT) excluding those common to all five genomes. Possible additional genes or gene fragments are given in brackets.

There are 16 copies of a conserved hypothetical protein - CHPmethylase in SLY. Annotated as putative methylases, (N-6 DNA methylase - Pfam02384), they are either 209 or 225 aa in length, except for three copies which are truncations. They are very highly conserved, with the putative full length proteins having 82% - 100% identity over the common 209 aa stretch. There are nine copies in PAa, but no apparent orthologues in OY-M, AY-WB or AT. They have, however, 51% - 58% identity to a hypothetical protein from *Spiroplasma citri* (CAL0075), the only match found in any other mollicute. These putative methylases are associated with PMUs in all but two cases (SLY352 and SLY1012). SLY352 is a truncation having only the first 45 aa at the amino terminus, while SLY1012 is a 209 aa protein with 97% - 98% identity with the other 209 aa paralogues. There is only one copy of llaDCHIA in PAa (PA0250) where it is annotated as *dam* “Site-specific DNA methylase”. This has 90% - 97% identity at an amino acid level with the 11 full-length proteins in SLY (two are truncations). There do not appear to be orthologues to this protein in any of the other published phytoplasma genomes. The closest match outside of ‘*Ca*. Phytoplasma australiense’ is to three putative methylases in ‘*Ca*. Phytoplasma asteris’. These ORFs are AYWB_379, PAM409, and PAM565, are annotated as “*dam* methylase”, and the percentage identity is much lower, at 38% - 40%. PA0438 is annotated as “restriction enzyme alpha subunit” and is located in a PMU between blocks “H” and “I”. No orthologue is detected in SLY; however, a BLASTp search revealed a 38% identity with apple proliferation phytoplasma genes ATP_00026 and ATP_00472, which are annotated as N-6 DNA methylases.

**Figure 5:**
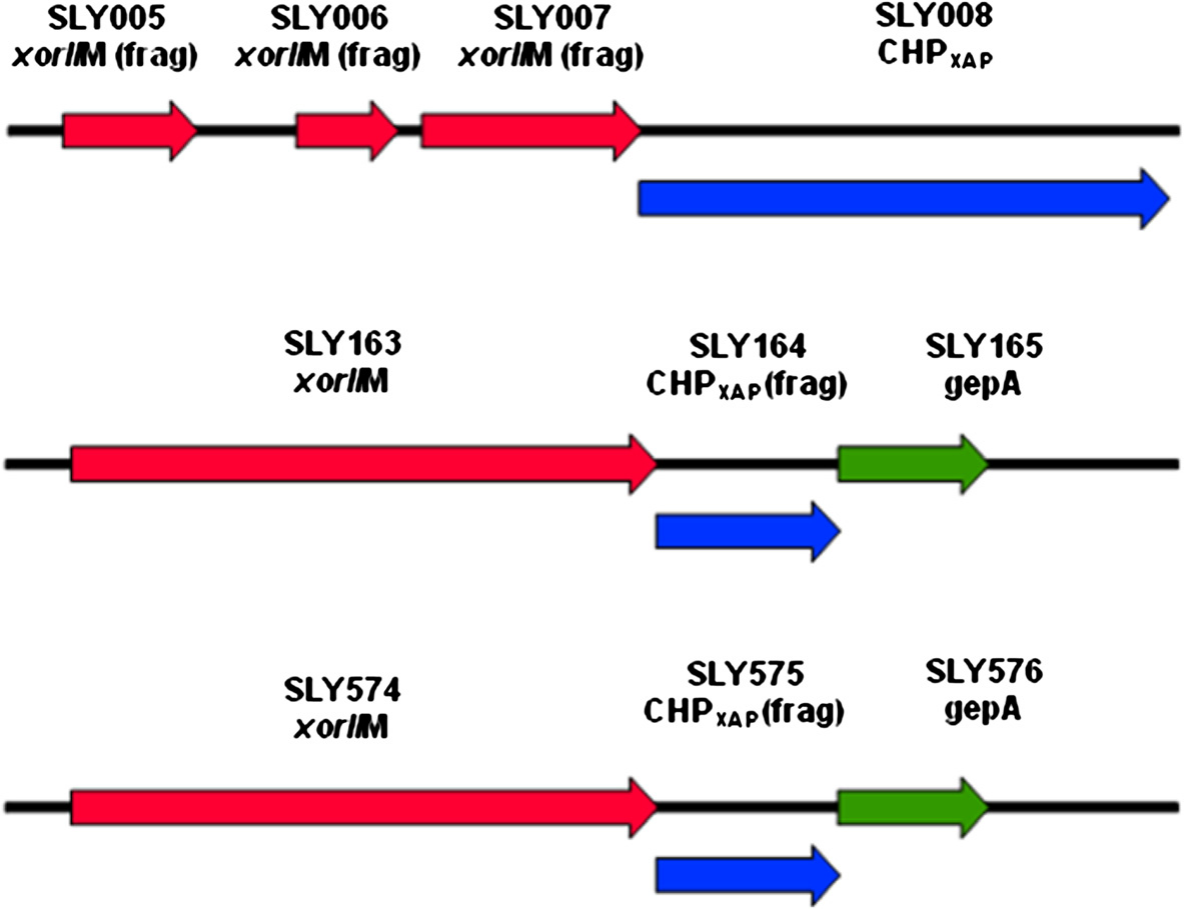
**Graphical depiction of *xor*IIM and CHP**_**xap **_**in SLY.** Graphical depiction of genes and fragments of *xor*IIM and CHP_xap_ from three locations on the SLY genome. SLY164 and SLY575 are truncations with the Open Reading Frames interrupted by putative phage related protein (*gepA*).

There are ten ORFs in SLY that are annotated as *xor*IIM. Five copies consist of a 418 amino acid protein and five ORFs that are fragments. This gene is very highly conserved, with the full-length copies differing at only 3/1257 nucleotides, which results in two amino acid changes (99.5% identity). It is annotated as a putative methylase, and the predicted protein has 46% identity to that of *Helicobacter acinonychis* str. Sheeba (YP_664125). Another ORF for which the closest match is to a *Helicobacter acinonychis* str. Sheeba ORF (YP_664126) is SLY008 (35% identity), coding for a conserved hypothetical protein 378 aa in length. There are two other ORFs in the SLY (SLY164 and SLY575) that are truncations of this protein, and they both also follow ORFs that code for *xor*IIM (SLY007 is a truncation of *xor*IIM) (Figure 5). Like *xor*IIM, the putative amino acid sequence for this CHP is very highly conserved, and differs at only 1 out of 125 aa residues (at the N-terminus), and the two fragments are identical to each other at a protein level. The 3′ end of SLY007 (*xor*IIM fragment) overlaps the 5′ end of SLY008 by 24 nucleotides, which suggests that these two genes were possibly part of an operon. Subsequently we have termed SLY008 and paralogues: CHPxap (for *xor*IIM-associated conserved hypothetical protein). Two fragments of *xor*IIM are present in PAa (PA0003 and PA0004) as consecutive ORFs, which together cover the amino and carboxy termini of the putative complete gene. CHPxap occurs as ORF PA0005. PA0005 is truncated when compared with the SLY orthologue (334 aa v. 378 aa), and differs by only 1 aa over that length. The two truncations of CHPxap in SLY are part of an exact (presumably recent) duplication. In SLY it seems as though *xor*IIM has been duplicated by being incorporated into a PMU. The accompanying ORF CHPxap has only become partially incorporated and as a result the complete ORF has not been successfully duplicated. Because *xor*IIM and CHPxap are highly conserved in SLY and PAa, and these genes have not been reported in any other firmicute, the possibility that these genes have been acquired by lateral (horizontal) gene transfer after the divergence of ‘*Ca*. Phytoplasma australiense’ and ‘*Ca*. Phytoplasma asteris’ is discussed below.

### Other genes involved in genome plasticity

As noted above, with the exception of *ltrA* almost all genes repeated more than 6 times in SLY are associated with PMUs. PAa, SLY and OY-M code for ORFs annotated as *ltrA*, an intron II reverse transcriptase. SLY has eight ORFs annotated as *ltrA*, although five appear to be fragments of the three longer versions (SLY039, SLY051 and SLY814). PAa has 16 ORFs annotated as retron-type reverse transcriptase, although all appear to be truncations of the full length gene. At five locations the fragments are consecutive, because of in-frame stops caused by INDELs or single nucleotide polymorphisms (SNPs).

The single orthologue in OY-M is PAM342, and two fragments have been reported from ‘*Ca*. Phytoplasma solani’: CAJ17913 (278 aa) and CAJ17954 (278 aa). The fragments from ‘*Ca*. Phytoplasma solani’ have 93% - 94% identity to that of PAM342, but only 71% identity with SLY814 over that same area. No apparent orthologues have been reported in the other published mollicute genomes, although this gene appears to be present in other firmicutes such as the Clostridia and Lactobacilli. Unlike other putative transposable elements found in SLY, *ltrA* does not appear to be closely linked to the PMUs, with only 2 of the 8 ORFs being within a PMU region. However, the gene appears to be associated with some genome rearrangements. SLY051 (*ltrA*), for example, appears to split a *tra5* gene in two. When the 2496 bp insert is removed (*in silico*), the *tra5* has a restored ORF. In PAa, a 30379 bp PMU is inserted in the orthologue of SLY746 (PA0212). Between PAa and SLY, the 607 bases of the 5′ UTR and c. 143 bases of the 3′ UTR of *ltrA* have a high degree of conservation (see Additional file 9), which may give an indication of the mode of action of this putative retron-type reverse transcriptase.

The putative peptides produced by the three *recA* fragments (SLY1046-1048) constitute a protein that matches the complete *recA* of *Acholeplasma laidlawii* (ACL_0890). A putative full-length *recA* is present in apple proliferation phytoplasma (ATP_00135). The preservation of these fragments may indicate that a functional *recA* protein is produced, and that these are programmed frameshifts. In SLY the putative complete ORF is interrupted by a single base deletion before the end of the first fragment, and a single base insertion before the end of the second fragment. The putative complete ORF in OY-M is disrupted by single base mutations that introduce two additional in-frame stops. The two putative full length phytoplasma *recA*s have 66% identity to each other at an amino acid level, and 58% to that of *A. laidlawii*. When compared with AT *recA*, the identities are OY-M 63%, SLY 61%, and *A. laidlawii* 60%.

## Discussion

SLY is the largest phytoplasma genome sequenced to date, with the variation in size due to the number and size of PMUs. This is evident in the comparison of PAa and SLY, two isolates of ‘*Ca*. Phytoplasma australiense’. A comparison of 23 genes not associated with PMUs, reveals a difference between the two isolates of less than 1% at a nucleotide level. By contrast, a comparison of similar genes between OY-M and AYWB (both members of ‘*Ca*. Phytoplasma asteris’) reveals differences of at least 4% - 5%. This very close phylogenetic relationship gives a unique insight into the variation occurring over shorter times of divergence in phytoplasma genomes, and thus provides additional insight into the evolution of these unique mollicutes.

The differences between SLY and PAa are not restricted to the number and size of PMUs; there is also some variation in synteny. It is possible that a measure of changed gene order is due to misassembly of the sequencing data. The large amount of repeated sequences posed considerable problems in the assembly of SLY. This is reflected in the fact that 73 clones from the 8–10 kb insert library needed to be completely sequenced before the final assembled contig could be established. However, identification of sequences that are unique to each isolate suggests that there are differences between the two genomes that could not simply be the result of one genome being misassembled.

There appear to be three main causes of plasticity in the ‘*Ca*. Phytoplasma australiense’ genomes. The first is PMUs, the second is centred on the putative retrotransposon, *ltrA*, and the third is the acquisition of new genetic material.

### Lateral gene transfer

In our analysis of SLY, we have found an example of recent acquisition of genetic material that is most easily explained by lateral gene transfer (LGT). ORFs SLY005-007 are fragments of a putative methylase - *xor*IIM, and the closest match by BLASTp search was to *Helicobacter acinonychis* str. Sheeba (YP_664125). The next putative ORF in SLY (SLY008), which we labelled CHPxap, codes for a CHP, with the closest match being the next ORF in *Helicobacter acinonychis* str. Sheeba (YP_664126). These two ORFs have not been detected in any firmicutes other than ‘*Ca.* Phytoplasma australiense’, so consequently we deduce that this acquisition has occurred after the separation of ‘*Ca*. Phytoplasma australiense’ and ‘*Ca*. Phytoplasma asteris’. It is interesting to note that in SLY, of these two ORFs only the putative methylase seems to have become incorporated into the PMUs and replicated six more times, whilst the CHPxap appears twice more as amino terminal fragments only. Furthermore, only one copy of *xor*IIM and CHPxap occurs in PAa and this is outside a PMU. Consequently we interpret this as being the likely site of initial introduction. In PAa, however, these ORFs have not been incorporated into a PMU for further dissemination. If this scenario is correct, then this also gives an indication of how quickly an ORF can be duplicated once it is incorporated into a PMU.

### Retrotransposon

The gene *ltrA* is annotated as intron II reverse transcriptase. In ‘*Ca*. Phytoplasma australiense’ one copy of this gene (SLY051) appears to be inserted into a *tra5* gene – a putative insertion sequence (IS) element. This corresponds with the characteristics of bacterial group II introns in that they are inserted either between genes, or in mobile DNAs, such as IS elements or plasmids [31]. When a 2496 bp fragment was removed *in silico*, a putative complete *tra5* protein sequence was restored. The inserted sequence comprised 607 bp 5′ UTR, 1746 bp ORF, and 143 bp 3′ UTR. This sequence was highly conserved in ‘*Ca*. Phytoplasma australiense’, and may provide an insight into the mechanism by which this sequence inserts into the phytoplasma genome. Two mechanisms are reported by which *ltrA* operates, one of which requires *recA*[32]. While the putative genes *ltrA* and three *recA* fragments are present in OY-M and SLY, they are absent in AY-WB. The three *recA* fragments are also missing from PAa, but this loss may be a recent event, as evidenced by the fact that PAa and SLY are very close phylogenetically, and the remnant is still present in OY-M. This could explain why there does not appear to be full length *ltrA* in PAa. Possibly full length copies stopped being duplicated or reincorporated with the absence of *recA*, and the existing copies are accumulating errors and are in the process of being eliminated. If correct, then this may indicate that SLY and OY-M produce a functional *recA* from three ORF fragments by a mechanism such as ribosome slippage. Translational frameshifting has been reported for Insertion Sequence family IS*3,* where the sequence can be divided between two overlapping reading frames OrfA and OrfB and a fusion protein produced by a −1 translational frameshift [33,34]. The presence in SLY of at least two IS-like elements belonging to the IS*3* family (annotated in SLY as *tra5*) with ORFs that seem to be arranged in this manner indicates that phytoplasmas possibly possess such a mechanism.

### PMUs

By far the greatest influence on genome variation in ‘*Ca*. Phytoplasma australiense’ is the PMU, which we estimated constituted 39% of the SLY genome. Bai *et al*. [19] identified clusters of genes in AY-WB and OY-M that were repeated throughout the genome. The presence of insertion sequences and other genes involved in recombination and repair strongly suggested that these clusters were mobile, and thus they were named potential mobile units (PMUs) [19]. When we further examined the composition of these gene clusters, we discovered that within each PMU there is an arrangement of three ORFs, consisting of a DNA helicase (*dnaC*), a thymidylate kinase (*tmk-a*), and a conserved hypothetical protein that appears to be associated with *tmk-a* (CHPtap), which extends the identification of conserved PMU genes by Arashida *et al.*[25]. In SLY, the order of these genes is *dnaC*, CHPtap and *tmk-a*. This contrasts with the analogous gene set in AY-WB, which is orientated in the reverse order *tmk-a*, CHPtap and *dnaB*, followed by an additional gene – *dnaG* (DNA primase). Both OY-M and PAa have examples of both types of PMUs, while AY-WB has just the one type. SLY possesses only one type, although it does have a solitary *dnaB* that appears to be a remnant of the AY-WB-type. It would appear therefore that there were at least two types of PMUs present in the last common ancestor of ‘*Ca*. Phytoplasma australiense’ and ‘*Ca*. Phytoplasma asteris’. This supports the proposal of Jomantine *et al.*[22] that these multi-gene clusters were introduced early in the development of the Phytoplasma clade. It also appears that some form of competition has occurred, resulting in one type become predominant within each isolate. This presumably reflects the contribution of PMUs to the fitness of the phytoplasma by acquiring or developing genes that endow the mollicute with extra capability. This could be through propagation of new genetic material that may be introduced by LGT, of which *xor*IIM may be an example, or by creation of new genes by means of deletion, fusion and rearrangement.

### Methylases

A further difference between SLY and PAa is the type and number of putative methylases present. In PAa, CHPmethylase (PA0043 and paralogues) is the dominant methylase with 12 copies, although 7 copies of a putative methylase (PA0253 and paralogues) were detected, of which 5 appear to be fragments of the putative full-length protein. Otherwise there are 4 other putative modification methylases annotated as *llaDCHIA* (PA0250), *xor*IIM (PA0003 and PA0004), hpaIM (PA0655 and PA0722), and “restriction enzyme alpha subunit” (PA0438) that are present in 1 or 2 copies. In SLY, at least 5 types were detected: CHPmethylase (13 copies), *llaDCHIA* (13 copies), *hpa*IM (2 copies), a fragment that has similarity with PA0253, and *xor*IIM. Although *xor*IIM was present in PAa, it was not associated with a PMU. No analogue of PAa gene (PA0438), annotated as a restriction-modification enzyme “restriction enzyme alpha subunit”, is present in SLY.

At least one putative methylase is present in each SLY PMU except for PMU5 and PMU6. SLY-PMU5 was identified as an abnormal PMU, as the *dnaC* was spread over two ORFS, and the *tmk-a* was truncated to the extent that it does not register as an ORF. SLY-PMU6 could be undergoing the same process. It is the smallest of all the putative PMUs, encompassing a mere 6851 bp, and the *tmk-a* for this PMU (SLY466) is severely truncated.

Methylases are associated with a number of functions in prokaryotes including nucleotide mis-match repair, DNA protection and gene regulation and control. Methylation has also been associated with transposition, so it is not surprising that they are closely associated with PMUs. Of the putative genes that occur in the phytoplasma genomes released to date, methylases appear to be the most diverse group, particularly those associated with PMUs. Apart from *yhhF* (PAM299), which appears to have orthologues in all phytoplasma genomes, the other methylases tend to be specific to the Candidatus species (Table 4). It is not known if these proteins are functionally different, but if they are, then perhaps they play a role in producing the distinctive symptomology associated with each organism. Even if the primary consequence of methylases in phytoplasmas has been to assist in the perpetuation of PMUs, a secondary effect could be to alter the methylation of plant host genes, thereby causing developmental effects that are characteristic of phytoplasma-associated diseases. This could require these proteins to be exported from the mollicute into the host. Alternatively, another mechanism such as RNAi could be activated and genes involved with the methylation of plant host genes could be silenced. In this way, PMUs could resemble pathogenicity islands present in other pathogenic bacteria.

Analysis of the two isolates of ‘*Ca*. Phytoplasma australiense’ revealed the 5′ UTR of the putative gene *rpoD* to be very highly conserved. The 5′ UTR is an area associated with gene regulation and control, and so a high degree of conservation would be expected. In ‘*Ca*. Phytoplasma australiense’, however, it seemed to present a discrete boundary associated with genome rearrangements. In SLY, it marked the start of a c. 12 kbp exact repeat, and there are two occasions where an ORF has been altered as a result of the intrusion of the 5′ UTR of an *rpoD* gene. This strongly indicates that the ORF disruption is the result of an insertion event, with the 5′ UTR of *rpoD* being one of the boundaries of the inserted sequence. It has also featured as distinct junctions in ‘*Ca*. Phytoplasma australiense’ where the sequences of the two isolates differ. This supports our belief that these highly conserved sequences represent one boundary of these mobile units, and may help us to understand how PMUs replicate throughout the genome. That only one boundary is discrete may reflect how the transposed sequence is integrated. Presumably the discrete boundary reflects the origin of insertion, perhaps as a result of a double stranded break (DSB) and ligation reaction, analogous to the asymmetric T-DNA insertions as a result of *Agrobacterium tumefaciens*-mediated plant transformations [35].

The inability to culture phytoplasmas axenically has undoubtedly hindered research into these important plant pathogens. Despite this handicap, much is being discovered about these organisms, with genomics leading the way. As more and more genomes are published, it is anticipated that our understanding of the biology of phytoplasmas, including how they cause pathogenicity, will increase, eventually leading to robust methods for their control or elimination. Such methods may involve targeting key phytoplasma-specific genes, or genes involved in pathogenicity using microRNAs.

## Conclusions

The complete assembled sequences of two closely related ‘*Ca*. Phytoplasma australiense’ genomes has provided an opportunity to understand the dynamic nature of phytoplasma chromosomes. Although the housekeeping genes of the two isolates differ by less than 1% at a nucleotide level, the genomes vary considerably in size and in their syntenic organisation. This genome plasticity is caused by several factors, including the presence of a putative retrotransposon *ltrA*. One mechanism for *ltrA* to operate requires a functional *recA*, which might indicate that the *recA* gene, which in SLY is fragmented by two INDELs, might actually produce a functional protein by a mechanism such as ribosomal slippage.

The main cause of variation between PAa and SLY is due to the proliferation of PMUs – clusters of genes that are adept at spreading through the genome. In this paper we have identified a number of conserved genes and gene arrangements that appear to be core to these PMUs. Comparison of these core genes and their syntenic arrangement also leads us to conclude that there are at least two species of PMUs in ‘*Ca*. Phytoplasma’. One key feature of these units, and one that possibly contributes to their fitness, is the acquisition of methylases. Our analysis indicated that the complement of methylases seemed distinctive to each ‘*Ca*. Phytoplasma’ species. The putative methylase *xor*IIM provided a possible explanation of how this occurs.

We believe that *xor*IIM was acquired by ‘*Ca*. Phytoplasma australiense’ by lateral gene transfer. In SLY it had been incorporated into a PMU and disseminated widely throughout the genome, whereas in PAa it remained as a single copy outside a PMU.

We also identified a conserved area of the 5’ UTR of the *rpoD* gene that seems to be associated with a discrete boundary of the PMUs. This should provide a guide to the mechanism of how these elements replicate and integrate into the genome.

Identification of such factors could only be done by comparison of assembled genomes, and is vindication of the effort that went into ensuring that that occurred. The development of next generation sequencing will provide the opportunity for many more genomes to be sequenced in a timely and cost-efficient manner. Some of the benefits of that data will only come about if there are robust assembled genomes for comparison, together with an understanding of how rearrangements occur. Identification of units or components of the genome that are involved in genomic reorganisation, such as have been described in this paper, will assist with this next phase of phytoplasma genomic studies.

## Methods

### Phytoplasma isolate

The isolate NZSb11 originated from a single strawberry plant displaying strawberry lethal yellows symptoms from a commercial garden in Katikati, Bay of Plenty, New Zealand. PCR amplification and sequencing of the 16S rRNA and *tuf* genes revealed that it belonged to 16SrXII-B and *Tuf* gene clade 1 [15,36,37].

### Purification of genomic DNA

Total genomic DNA was extracted from stolons and petioles from symptomatic strawberry runners by the CTAB procedure of Doyle and Doyle [38]. The phytoplasma and plant host DNA were separated by three rounds of bisbenzimide-CsCl buoyant density gradient centrifugation as described by Kollar *et al*. [39]. This technique utilises the principle that bisbenzimide dye binds preferentially to A + T-rich sequences of the phytoplasma, thereby reducing their buoyant density and allowing separation from the higher G + C content of the host DNA on equilibrium density gradients.

### Genome sequencing and assembly

The complete genome sequence of ‘*Ca*. Phytoplasma australiense’ was determined using the whole-genome shotgun method. Two small-insert plasmid libraries (2–3 kb and 3–4 kb) were constructed in pUC18 and one medium-insert plasmid library (8–10 kb) was constructed in a low copy number vector, pMCL200 [40] using mechanically sheared DNA. DNA sequencing of the insert ends was carried out using BigDye 3.1 terminator chemistry and resolved with automated capillary ABI3700 sequencers. In the initial random-sequencing phase, 9-fold sequence coverage was achieved from the three libraries (3.3-fold coverage for each of the two small-insert libraries and 2.4-fold coverage for the medium-insert library). Sequences were assembled into contigs using the PHRAP assembly tool [41]. The assembly was visualised and edited using GAP4 [42] of the STADEN package software [43]. To solve problems with misassembled regions caused by repetitive sequences and to close remaining sequence gaps, 6 clones from the 3–4 kb insert library and 73 clones from the 8–10 kb insert library were completely sequenced by primer walking. The assembly was repeated, incorporating the backbones from these individually assembled primer-walked clones along with the shotgun reads. Regions of single-clone coverage and low sequence quality were resequenced by primer walking on clones from the small-insert plasmid libraries. The coverage criterion was that every base was covered by at least two clones of high-quality sequence in each direction. The final assembly contained ~16,975 shotgun and primer walk reads, giving 10-fold coverage of the genome.

### Annotation and analysis

Annotation was performed by BASys, an automated pipeline that combines GLIMMER gene prediction, ORF and non-ORF feature identification, and assignment of functional role categories to genes [44]. Paralogous gene families were defined using a cut-off E value of 10^-5^ with at least 60% query coverage and 50% identity. An all-v.-all BLASTP search was performed with the protein set to identify pairwise matches above E ≤ 10^-10^ over ≥80% coverage. Putative coding sequences were considered for peptides of 30 aa or greater. Unless otherwise stated, protein similarity refers to % identity.

### Comparative genomics

Phytoplasma genome sequences OY-M [GenBank NC_005303], AYWB [GenBank CP000061], PAa [GenBank AM422018] and AT (Apple proliferation) [GenBank CU469464] were obtained from GenBank. All NZSb11 ORFs were compared by BLASTP against the complete set of ORFs from OY-M, AY-WB and PAa, and each ORF from OY-M, AY-WB and PAa was compared using BLASTP against the annotated NZSb11 (SLY) genome. Searches were conducted using NCBI database or Molligen (http://cbi.labri.fr/outils/molligen/) [45].

Sequence analysis was conducted using VectorNTI® 9.0.0 (Life Technologies, Carlsbad, CA) suite of programs or Sequencher™ 4.9 (Gene Codes Corporation, Ann Arbour, MI).

The complete annotated genome sequence is available from GenBank, accession number CP002548.

## Competing interests

The authors declare that they have no competing interests.

## Authors’ contributions

DNA isolation and sequencing was done by LWL. Assembly and primary annotation by LWL and IH. More detailed annotation and manuscript preparation by MTA. The project was managed by REB. All authors read and approved the final manuscript.

## Supplementary Material

Additional file 1 **Open Reading Frames (ORFs) of SLY genome.** List of Open Reading Frames (ORFs) of SLY genome predicted to be protein coding genes, with putative annotation. Click here for file

Additional file 2 **Potential Mobile Units (PMUs) in the SLY genome.** Graphical representation of size and distribution of areas consisting of Potential Mobile Units (PMUs) in the SLY genome. Click here for file

Additional file 3 **Potential Mobile Units (PMUs) of PAa.** Size of PAa Potential Mobile Units (PMUs) as determined using Open Reading Frame (ORF) boundaries. Boundaries were determined by the association of ORFs with PMUs, including hypothetical proteins (HP) and conserved hypothetical proteins (CHP). Click here for file

Additional file 4 **Comparison of a selection of “housekeeping” genes of PAa and SLY and OY-M and AY-WB.** DNA comparison of a selection of “housekeeping” genes of two ‘*Candidatus* Phytoplasma australiense’ isolates PAa and SLY (A) and two ’*Ca*. Phytoplasma asteris’ isolates OY-M and AY-WB (B). Most genes were present in each genome as a single copy. Two copies of *mtgA* were present in OY-M and AY-WB, and those with comparable syntenic positions were compared. Click here for file

Additional file 5 **Composition of ‘*Candidatus *Phytoplasma australiense’ *rpoD *Groups A and B.** SLY570 lacks a 5′ UTR because of the insertion of SLY571, and cannot be placed in GpA or GpB, although the amino acid sequence aligns better with GpA samples than those belonging to GpB. Click here for file

Additional file 6 **Example of an SLY Open Reading Frame (ORF) being altered by the intrusion of the 5′ Untranslated Region (UTR) of the *rpoD *gene.** (A) Diagram illustrating that the 150 bp 5′ UTR of SLY1027 (*rpoD* Group B) overlaps with the 3′ region of the ORF SLY1026. (B) A line-up of predicted amino acid sequences of SLY1026 and paralogues shows a high degree of sequence identity except for 11 residues of the carboxyl (3′) terminus. (C) Nucleic acid alignment of SLY1026 and paralogues shows that the point at which sequences differ corresponds to the 5′UTR of SLY1027 (bottom row). Yellow colouring indicates exact nucleotide match and blue colouring is greatest consensus match. Click here for file

Additional file 7 **Junctions associated with 5′ Untranslated Region of *rpoD*.** Junctions associated with 5′ Untranslated Region of *rpoD* of ‘*Candidatus* Phytoplasma australiense’ isolates PAa and SLY. Click here for file

Additional file 8 **Putative modification methylases in** ‘***Candidatus *Phytoplasma australiense’.** Putative modification methylases in ‘*Ca*. Phytoplasma australiense’ isolates PAa and SLY. Click here for file

Additional file 9 **Conserved sequences associated with *ltrA *in ‘*Candidatus *Phytoplasma australiense’.** Phytoplasma australiense’. Yellow colouring indicates exact nucleotide match and blue colouring is greatest consensus match. (A) Graphical representation of the 5′ Untranslated Region (UTR) (607 bp), Open Reading Frame (1746 bp) and 3′ UTR (143 bp) of SLY; (B) line-up of the approximately the 5′ most 90 bp of the 5′ UTR showing the boundary of the conserved sequence; (C) alignment of c. 150 bp of the 3′ UTR. Lowest sequence of the alignment represents the 3′ end of the *ltrA* ORF. Click here for file
