# Supplementary material for: Comparison of the complete genome sequence of two closely related isolates of ‘Candidatus Phytoplasma australiense’ reveals genome plasticity
Source: BMC Genomics. 2013 Aug 2;14:529. doi: 10.1186/1471-2164-14-529 (PMC3750655; doi:10.1186/1471-2164-14-529)
Supplement: Additional file 1 — Open Reading Frames (ORFs) of SLY genome. List of Open Reading Frames (ORFs) of SLY genome predicted to be protein coding genes, with putative annotation. [file 1471-2164-14-529-S1.pdf]

Additional file 1

Table S1. List of Open Reading Frames (ORFs) of SLY genome predicted to be protein coding genes, with putative annotation.

ORF	Gene	Protein Function
SLY001	*dnaA*	Chromosomal replication initiation protein
SLY002	*dnaN*	DNA polymerase III beta subunit
SLY003	-	Hypothetical Protein
SLY004	-	Hypothetical Protein
SLY005	*xor*IIM	Modification methylase XorII (fragment)
SLY006	*xor*IIM	Modification methylase *Xor*II (fragment)
SLY007	*xor*IIM	Modification methylase *Xor*II (fragment)
SLY008	CHP	Conserved Hypothetical Protein associated with *xor*IIM
SLY009	*ykqC*	Metallo-Beta-Lactamase Family Protein
SLY010	*degV*	*DegV* Family Protein
SLY011	*ybjI*	Hydrolase Haloacid Dehalogenase-Like Family (fragment)
SLY012	*ybjI*	Hydrolase Haloacid Dehalogenase-Like Family (fragment)
SLY013	*ybjI*	Hydrolase Haloacid Dehalogenase-Like Family
SLY014	*ykqC*	Metallo-Beta-Lactamase Family Protein
SLY015	*spxA*	Regulatory protein *spx*
SLY016	*artM*	ABC Transporter Permease Component (fragment)
SLY017	*yecS*	Inner membrane amino-acid ABC transporter permease protein yecS
SLY018	CHP	Conserved Hypothetical Protein
SLY019	-	Hypothetical Protein
SLY020	-	Hypothetical Protein
SLY021	-	Hypothetical Protein
SLY022	-	Hypothetical Protein
SLY023	*thrS*	Threonyl-tRNA synthetase
SLY024	CHP	Putative Exopolyphosphatase-related protein
SLY025	*trmE*	tRNA modification GTPase trmE
SLY026	CHP	Mgp-operon protein 1
SLY027	*MalE*	Putative maltose/maltodextrin-binding protein
SLY028	*ycjP*	Probable ABC transporter permease protein MG189
SLY029	*ugpA*	Probable ABC transporter permease protein MG188
SLY030	*fbpC*	Ferric cations import ATP-binding protein *fbpC*
SLY031	*asnS*	Asparaginyl-tRNA synthetase
SLY032	-	Hypothetical Protein
SLY033	*tra5*	Putative transposase *tra5* for insertion sequence element IS150
SLY034	*tra5*	Putative transposase *tra5* for insertion sequence element IS150
SLY035	-	Hypothetical Protein
SLY036	-	Hypothetical Protein
SLY037	CHP	Conserved Hypothetical Protein
SLY038	CHP	Conserved Hypothetical Protein
SLY039	*ltrA*	Group II intron-encoded protein *ltrA* [Includes: Reverse-transcriptase
SLY040	-	Hypothetical Protein
SLY041	CHP	Conserved Hypothetical protein - Paragroup CHP041
SLY042	*dnaC*	Replicative DNA helicase
SLY043	*tra5*	Putative transposase *tra5* for insertion sequence element IS150
SLY044	*rad50*	DNA double-strand break repair rad50 ATPase
SLY045	*yibP*	Hypothetical Protein *yibP* (fragment)
SLY046	*yibP*	Hypothetical Protein *yibP* (fragment)
SLY047	CHP	Conserved Hypothetical Protein
SLY048	CHP	Hypothetical protein -Paragroup CHP041
SLY049	-	Hypothetical Protein
SLY050	*tra5*	Putative transposase *tra5* for insertion sequence element IS150

ORF	Gene	Protein Function
SLY051	*ltrA*	Group II intron-encoded protein *ltrA* [Includes: Reverse-transcriptase
SLY052	*tra5*	Putative transposase *tra5* for insertion sequence element IS150
SLY053	*rpoD*	DNA-Directed RNA Polymerase Sigma Subunit
SLY054	CHP	Conserved Hypothetical Protein
SLY055	*dnaC*	Replicative DNA helicase
SLY056	CHP	Conserved Hypothetical Protein associated with *tmk*
SLY057	*tmk*	Thymidylate kinase
SLY058	llaDCHIA	Modification methylase LlaDCHIA
SLY059	CHP	Conserved Hypothetical Protein
SLY060	*hupB*	Hypothetical Protein hupB
SLY061	CHP	Conserved Hypothetical Protein (methylase)
SLY062	-	Hypothetical Protein
SLY063	-	Hypothetical Protein
SLY064	CHP	Conserved Hypothetical Protein
SLY065	-	Hypothetical Protein
SLY066	-	Hypothetical Protein
SLY067	CHP	Conserved Hypothetical Protein
SLY068	-	Hypothetical Protein
SLY069	*ftsH*	Cell division protein *ftsH* homolog
SLY070	*rad50*	DNA double-strand break repair *rad50* ATPase
SLY071	-	Hypothetical Protein
SLY072	-	Hypothetical Protein
SLY073	-	Hypothetical Protein
SLY074	*nrdE*	Ribonucleoside-diphosphate reductase alpha subunit
SLY075	-	Hypothetical Protein
SLY076	-	Hypothetical Protein
SLY077	*hupB*	Hypothetical Protein *hupB*
SLY078	-	Phage-Associated Protein
SLY079	*eno*	Enolase
SLY080	-	Hypothetical Protein
SLY081	-	Hypothetical Protein
SLY082	-	Hypothetical Protein
SLY083	*gpmI*	2,3-bisphosphoglycerate-independent phosphoglycerate mutase
SLY084	*pyk*	Pyruvate kinase
SLY085	-	Hypothetical Protein
SLY086	CHP	Conserved Hypothetical Protein
SLY087	-	Hypothetical Protein
SLY088	-	Hypothetical Protein
SLY089	CHP	Conserved Hypothetical Protein
SLY090	-	Hypothetical Protein
SLY091	-	Hypothetical Protein
SLY092	*thyA*	Thymidylate synthase
SLY093	*dfrA*	Dihydrofolate reductase
SLY094	-	Hypothetical Protein
SLY095	*plsC*	Probable 1-acyl-sn-glycerol-3-phosphate acyltransferase
SLY096	CHP	*OsmC/Ohr* Family Protein
SLY097	*rpsD*	30S ribosomal protein S4
SLY098	*mgtA*	Magnesium transporting ATPase, P-type 1
SLY099	CHP	Hypothetical protein MG105
SLY100	*degV*	degV Family Protein
SLY101	-	Hypothetical Protein
SLY102	-	Hypothetical Protein
SLY103	-	Hypothetical Protein
SLY104	*gcp*	Probable O-sialoglycoprotein endopeptidase
SLY105	-	Hypothetical Protein
SLY106	*dnaC*	Replicative DNA helicase
SLY107	*tra5*	Putative transposase *tra5* for insertion sequence element IS150
SLY108	*tra5*	Putative transposase tra5 for insertion sequence element IS150
SLY109	-	Hypothetical Protein
SLY110	CHP	Conserved Hypothetical Protein
SLY111	*yibP*	Hypothetical Protein *yibP* (fragment)

ORF	Gene	Protein Function
SLY112	*yibP*	Hypothetical Protein *yibP* (fragment)
SLY113	*rad50*	DNA double-strand break repair rad50 ATPase
SLY114	*ftsH*	Cell division protein *ftsH* homolog
SLY115	-	Hypothetical Protein
SLY116	CHP	Conserved Hypothetical Protein
SLY117	-	Hypothetical Protein
SLY118	CHP	Conserved Hypothetical Protein
SLY119	CHP	Conserved Hypothetical Protein
SLY120	CHP	Conserved Hypothetical Protein
SLY121	-	Hypothetical Protein
SLY122	CHP	Conserved Hypothetical Protein (methylase)
SLY123	*hupB*	Hypothetical Protein *hupB*
SLY124	CHP	Conserved Hypothetical Protein
SLY125	CHP	Phage-Associated Protein
SLY126	*tmk*	Thymidylate kinase
SLY127	CHP	Conserved Hypothetical Protein associated with *tmk*
SLY128	*dnaC*	Replicative DNA helicase
SLY129	CHP	Conserved Hypothetical Protein
SLY130	*rpoD*	DNA-Directed RNA Polymerase Sigma Subunit
SLY131	CHP	Hypothetical protein -Paragroup CHP041
SLY132	-	Hypothetical Protein
SLY133	CHP	Conserved Hypothetical Protein
SLY134	-	Hypothetical Protein
SLY135	*tra5*	Putative transposase *tra5* for insertion sequence element IS150
SLY136	*tra5*	Putative transposase *tra5* for insertion sequence element IS150
SLY137	-	Hypothetical Protein
SLY138	*ygiH*	Hypothetical UPF0078 membrane protein PAM436
SLY139	-	Hypothetical Protein
SLY140	*yqiY*	Probable amino-acid ABC transporter permease protein *yqiY*
SLY141	-	Hypothetical Protein
SLY142	*cadA*	Probable cadmium-transporting ATPase
SLY143	*spoVG*	Putative septation protein *spoVG*
SLY144	*ksgA*	Dimethyladenosine transferase
SLY145	*ywfO*	HD Domain Protein
SLY146	*pduL*	Propanediol Utilization Protein
SLY147	*tra5*	Putative transposase *tra5* for insertion sequence element IS150
SLY148	CHP	Conserved Hypothetical Protein
SLY149	CHP	Conserved Hypothetical Protein
SLY150	CHP	Hypothetical Protein
SLY151	CHP	Hypothetical protein -Paragroup CHP041
SLY152	CHP	Hypothetical Protein Paragroup CHP152
SLY153	*tra5*	Putative transposase *tra5* for insertion sequence element IS150
SLY154	*tra5*	Putative transposase *tra5* for insertion sequence element IS150
SLY155	CHP	Conserved Hypothetical Protein
SLY156	CHP	Conserved Hypothetical Protein
SLY157	CHP	Hypothetical protein -Paragroup CHP152
SLY158	CHP	Hypothetical protein -Paragroup CHP158
SLY159	CHP	Hypothetical protein -Paragroup CHP041
SLY160	*rpoD*	DNA-Directed RNA Polymerase Sigma Subunit
SLY161	-	Phage-Associated Protein
SLY162	-	Phage-Associated Protein
SLY163	*xor*IIM	Modification methylase *Xor*II
SLY164	CHP	Hypothetical Protein associated with *xor*IIM
SLY165	-	Phage-Associated Protein
SLY166	CHP	Conserved Hypothetical Protein
SLY167	*dnaC*	Replicative DNA helicase

ORF	Gene	Protein Function
SLY168	CHP	Conserved Hypothetical Protein associated with *tmk*
SLY169	*tmk*	Thymidylate kinase
SLY170	CHP	Conserved Hypothetical Protein
SLY171	llaDCHIA	Modification methylase LlaDCHIA
SLY172	CHP	Phage-Associated Protein
SLY173	CHP	Phage-Associated Protein
SLY174	CHP	Conserved Hypothetical Protein
SLY175	*ihfA*	Hypothetical Protein *ihfA*
SLY176	CHP	Conserved Hypothetical Protein (methylase)
SLY177	-	Hypothetical Protein
SLY178	-	Hypothetical Protein
SLY179	CHP	Conserved Hypothetical Protein
SLY180	CHP	Conserved Hypothetical Protein
SLY181	*hflB*	Putative cell division cycle ATPase
SLY182	CHP	Hypothetical protein -Paragroup CHP152
SLY183	CHP	Polyprotein -Paragroups CHP041 and CHP158
SLY184	CHP	Conserved Hypothetical Protein
SLY185	CHP	Conserved Hypothetical Protein
SLY186	*yibP*	Hypothetical Protein *yibP* (fragment)
SLY187	CHP	Conserved Hypothetical Protein
SLY188	CHP	Phage-Associated Protein
SLY189	-	Hypothetical Protein
SLY190	-	Hypothetical Protein
SLY191	CHP	Conserved Hypothetical Protein
SLY192	*tra5*	Putative transposase tra5 for insertion sequence element IS150
SLY193	CHP	Hypothetical protein -Paragroup CHP041
SLY194	-	Hypothetical Protein
SLY195	*pacL*	Cation-transporting ATPase pacL
SLY196	-	Hypothetical Protein
SLY197	*ileS*	Isoleucyl-tRNA synthetase
SLY198	*engD*	GTP-dependent nucleic acid-binding protein engD
SLY199	*engC*	Probable GTPase *engC*
SLY200	-	Hypothetical Protein
SLY201	-	Hypothetical Protein
SLY202	-	Hypothetical Protein
SLY203	*tra5*	Putative transposase *tra5* for insertion sequence element IS150
SLY204	*tra5*	Putative transposase *tra5* for insertion sequence element IS150
SLY205	*hflB*	Putative cell division cycle ATPase
SLY206	CHP	Conserved Hypothetical Protein
SLY207	-	Hypothetical Protein
SLY208	CHP	Conserved Hypothetical Protein
SLY209	CHP	Conserved Hypothetical Protein
SLY210	-	Hypothetical Protein
SLY211	CHP	Conserved Hypothetical Protein (methylase)
SLY212	CHP	Conserved Hypothetical Protein (methylase)
SLY213	-	Hypothetical Protein
SLY214	*ihfA*	Hypothetical Protein ihfA
SLY215	CHP	Conserved Hypothetical Protein
SLY216	-	Hypothetical Protein
SLY217	CHP	Conserved Hypothetical Protein
SLY218	llaDCHIA	Modification methylase LlaDCHIA
SLY219	CHP	Conserved Hypothetical Protein
SLY220	*tmk*	Thymidylate kinase
SLY221	CHP	Conserved Hypothetical Protein associated with *tmk*
SLY222	*dnaC*	Replicative DNA helicase
SLY223	*rad50*	DNA double-strand break repair *rad50* ATPase
SLY224	*yibP*	Hypothetical Protein yibP (frag)
SLY225	-	Hypothetical Protein
SLY226	-	Hypothetical Protein
SLY227	CHP	Conserved Hypothetical Protein
SLY228	*tra5*	Putative transposase *tra5* for insertion sequence element IS150

ORF	Gene	Protein Function
SLY229	*tra5*	Putative transposase *tra5* for insertion sequence element IS150
SLY230	*tra5*	Putative transposase *tra5* for insertion sequence element IS150
SLY231	CHP	Hypothetical Protein MCAP
SLY232	-	Hypothetical Protein
SLY233	*nfo*	Probable endonuclease IV
SLY234	*nfo*	Probable endonuclease IV
SLY235	*yqfR*	Probable ATP-dependent RNA helicase *yqfR*
SLY236	*ytmL*	Probable amino-acid ABC transporter permease protein ytmL
SLY237	*ribF*	Riboflavin biosynthesis protein ribF [Includes: Riboflavin kinase
SLY238	*uvrC*	UvrABC system protein C
SLY239	-	Hypothetical Protein
SLY240	-	Hypothetical Protein
SLY241	-	Hypothetical Protein
SLY242	*uvrB*	UvrABC system protein B
SLY243	CHP	Conserved Hypothetical Protein
SLY244	CHP	Conserved Hypothetical Protein
SLY245	*nifU*	*NifU* Homolog
SLY246	-	Hypothetical Protein
SLY247	CHP	Conserved Hypothetical Protein
SLY248	CHP	Conserved Hypothetical Protein
SLY249	CHP	Conserved Hypothetical Protein associated with tmk
SLY250	*dnaC*	Replicative DNA helicase
SLY251	*dnaC*	Replicative DNA helicase
SLY252	-	Hypothetical Protein
SLY253	CHP	Phage-Associated Protein
SLY254	CHP	Conserved Hypothetical Protein
SLY255	*rpoD*	DNA-Directed RNA Polymerase Sigma Subunit
SLY256	*rpoD*	DNA-Directed RNA Polymerase Sigma Subunit
SLY257	-	Hypothetical Protein
SLY258	CHP	Conserved Hypothetical Protein
SLY259	-	Hypothetical Protein
SLY260	-	Hypothetical Protein
SLY261	*tra5*	Putative transposase *tra5* for insertion sequence element IS150
SLY262	CHP	Conserved Hypothetical Protein
SLY263	-	Hypothetical Protein
SLY264	CHP	Conserved Hypothetical Protein
SLY265	-	Hypothetical Protein
SLY266	-	Hypothetical Protein
SLY267	*troA*	Periplasmic zinc-binding protein *troA* precursor
SLY268	*troB*	Zinc transport system ATP-binding protein *troB*
SLY269	*mntC*	Manganese transport system membrane protein *mntC*
SLY270	*troD*	Zinc transport system membrane protein *troD*
SLY271	*hisS*	Histidyl-tRNA synthetase
SLY272	*aspS*	Aspartyl-tRNA synthetase
SLY273	*tilS*	tRNA(Ile)-lysidine synthase
SLY274	*ftsH*	Cell division protein *ftsH* homolog
SLY275	*rluD*	Ribosomal Large Subunit Pseudouridine Synthase D
SLY276	*metN*	Probable D-methionine transport ATP-binding protein metN
SLY277	*nplA*	ABC-Type Methionine Transport System Periplasmic Component
SLY278	*metI*	D-methionine transport system permease protein metI
SLY279	*yaaQ*	Hypothetical protein *yaaQ*
SLY280	*rpsO*	30S ribosomal protein S15
SLY281	*thiJ*	Protein thiJ
SLY282	*rplM*	50S ribosomal protein L13
SLY283	*rpsI*	30S ribosomal protein S9
SLY284	*gltX*	Glutamyl-tRNA synthetase
SLY285	*cysS*	Cysteinyl-tRNA synthetase
SLY286	*yodC*	Putative NAD(P)H nitroreductase 12C

ORF	Gene	Protein Function
SLY287	CHP	Conserved Hypothetical Protein
SLY288	CHP	Hypothetical UPF0154 protein MYPE400
SLY289	*yhhF*	Putative methylase HI0767
SLY290	*yqeK*	Protein *yqeK*
SLY291	*yqeG*	Hypothetical protein *yqeG*
SLY292	*greA*	Transcription elongation factor *greA*
SLY293	*smtA*	O-Methyltransferase Family Protein
SLY294	*ruvX*	Putative Holliday junction resolvase
SLY295	*alaS*	Alanyl-tRNA synthetase
SLY296	-	Hypothetical Protein
SLY297	*gap*	Glyceraldehyde-3-phosphate dehydrogenase
SLY298	*pgk*	Phosphoglycerate kinase
SLY299	*fba*	Fructose-bisphosphate aldolase
SLY300	*yidA*	Peptidyl-Prolyl Cis-Trans Isomerase
SLY301	*tpiA*	Triosephosphate isomerase
SLY302	-	Hypothetical Protein
SLY303	-	Hypothetical Protein
SLY304	*cdsA*	Phosphatidate cytidylyltransferase
SLY305	*frr*	Ribosome recycling factor
SLY306	*pyrH*	Uridylate kinase
SLY307	*tsf*	Elongation factor Ts
SLY308	*rpsB*	30S ribosomal protein S2
SLY309	*dut*	Deoxyuridine 5'-triphosphate nucleotidohydrolase
SLY310	*ung*	Uracil-DNA glycosylase
SLY311	CHP	Conserved Hypothetical Protein
SLY312	-	Hypothetical Protein
SLY313	*pan*	Proteasome-activating nucleotidase
SLY314	-	Hypothetical Protein
SLY315	CHP	Conserved Hypothetical Protein
SLY316	*tra5*	Putative transposase tra5 for insertion sequence element IS150
SLY317	-	Hypothetical Protein
SLY318	-	Hypothetical Protein
SLY319	-	Hypothetical Protein
SLY320	-	Hypothetical Protein
SLY321	-	Hypothetical Protein
SLY322	-	Hypothetical Protein
SLY323	*lolD*	ABC transporter ATP-binding protein MJ0796
SLY324	*pheT*	Phenylalanyl-tRNA synthetase beta chain
SLY325	*pheS*	Phenylalanyl-tRNA synthetase alpha chain
SLY326	CHP	Conserved Hypothetical Protein
SLY327	*ybjI*	Hydrolase Haloacid Dehalogenase-Like Family (fragment)
SLY328	*ybjI*	Hydrolase Haloacid Dehalogenase-Like Family (fragment)
SLY329	*dnaE*	DNA polymerase III alpha subunit
SLY330	*lysS*	Lysyl-tRNA synthetase
SLY331	*lplA*	Probable lipoate-protein ligase A
SLY332	CHP	Conserved Hypothetical Protein
SLY333	*ftsH*	Cell division protein ftsH homolog
SLY334	*argS2*	Arginyl-tRNA synthetase 2
SLY335	*polC*	DNA polymerase III *polC*-type
SLY336	-	Hypothetical protein
SLY337	*trpS*	Tryptophanyl-tRNA synthetase
SLY338	-	Hypothetical Protein
SLY339	-	Hypothetical Protein
SLY340	-	Hypothetical Protein
SLY341	-	Hypothetical Protein
SLY342	*nifU*	NifU-like protein
SLY343	*csd*	Probable cysteine desulfurase
SLY344	*tyrS*	Tyrosyl-tRNA synthetase
SLY345	*rplS*	50S ribosomal protein L19
SLY346	*trmD*	tRNA (Guanine-1) methyltransferase
SLY347	*rpsP*	30S ribosomal protein S16
SLY348	*prfB*	Peptide chain release factor 2
SLY349	*secA*	Preprotein translocase *secA* subunit
SLY350	CHP	Conserved Hypothetical Protein
SLY351	-	Hypothetical Protein
SLY352	CHP	Conserved Hypothetical Protein (methylase)

ORF	Gene	Protein Function
SLY353	-	Hypothetical Protein
SLY354	*dnaJ*	Chaperone protein *dnaJ*
SLY355	*dnaK*	Chaperone protein *dnaK*
SLY356	*grpE*	Protein *grpE*
SLY357	*hrcA*	Heat-inducible transcription repressor *hrcA*
SLY358	-	Hypothetical Protein
SLY359	-	Hypothetical Protein
SLY360	*rplQ*	50S ribosomal protein L17
SLY361	*rpoA*	DNA-directed RNA polymerase alpha chain
SLY362	*rpsK*	30S ribosomal protein S11
SLY363	*rpsM*	30S ribosomal protein S13
SLY364	*rpmJ*	50S ribosomal protein L36
SLY365	*infA*	Translation initiation factor IF-1
SLY366	*map*	Methionine aminopeptidase
SLY367	*adk*	Adenylate kinase
SLY368	*secY*	Preprotein translocase *secY* subunit
SLY369	*rplO*	50S ribosomal protein L15
SLY370	*rpmD*	50S ribosomal protein L30
SLY371	*rpsE*	30S ribosomal protein S5
SLY372	*rplR*	50S ribosomal protein L18
SLY373	*rplF*	50S ribosomal protein L6
SLY374	*rpsH*	30S ribosomal protein S8
SLY375	*rpsN2*	30S ribosomal protein S14-2
SLY376	*rplE*	50S ribosomal protein L5
SLY377	*rplX*	50S ribosomal protein L24
SLY378	*rplN*	50S ribosomal protein L14
SLY379	*rpsQ*	30S ribosomal protein S17
SLY380	*rpmC*	50S ribosomal protein L29
SLY381	*rplP*	50S ribosomal protein L16
SLY382	*rpsC*	30S ribosomal protein S3
SLY383	*rplV*	50S ribosomal protein L22
SLY384	*rpsS*	30S ribosomal protein S19
SLY385	*rplB*	50S ribosomal protein L2
SLY386	*rplW*	50S ribosomal protein L23
SLY387	*rplD*	50S ribosomal protein L4
SLY388	*rplC*	50S ribosomal protein L3
SLY389	*rpsJ*	30S ribosomal protein S10
SLY390	*rpmF*	50S ribosomal protein L32
SLY391	-	Hypothetical Protein
SLY392	*obg*	Spo0B-associated GTP-binding protein
SLY393	*rpmA*	50S ribosomal protein L27
SLY394	*CHP*	Conserved Hypothetical Protein
SLY395	*rplU*	50S ribosomal protein L21
SLY396	*yceA*	Rhodanese-Like
SLY397	*serS*	Seryl-tRNA synthetase
SLY398	-	Hypothetical Protein
SLY399	-	Hypothetical Protein
SLY400	*rpsT*	30S ribosomal protein S20
SLY401	-	Hypothetical Protein
SLY402	-	Hypothetical Protein
SLY403	*NUF1*	Protein NUF1
SLY404	-	Hypothetical Protein
SLY405	*thiI*	Probable thiamine biosynthesis protein thiI
SLY406	*ppa*	Inorganic pyrophosphatase
SLY407	*smpB*	SsrA-binding protein
SLY408	-	Hypothetical Protein
SLY409	-	Hypothetical Protein
SLY410	-	Hypothetical Protein
SLY411	CHP	Hypothetical Protein Pcar
SLY412	*nusA*	Transcription elongation protein nusA
SLY413	CHP	Conserved Hypothetical Protein
SLY414	*infB*	Translation initiation factor IF-2
SLY415	*rbfA*	Ribosome-binding factor A
SLY416	*pth*	Peptidyl-tRNA hydrolase
SLY417	*yhdP*	Hemolysin
SLY418	CHP	Hypothetical Protein MSC
SLY419	*sigA*	RNA polymerase sigma factor *rpoD*
SLY420	*dnaG*	DNA primase
SLY421	*glyQS*	Glycyl-tRNA synthetase
SLY422	*era*	GTP-binding protein era homolog
SLY423	*ybeY*	Hypothetical UPF0054 protein
SLY424	-	Hypothetical protein
SLY425	*rpsU*	30S ribosomal protein S21
SLY426	-	Hypothetical Protein
SLY427	CHP	Conserved Hypothetical Protein
SLY428	CHP	Conserved Hypothetical Protein
SLY429	-	Hypothetical Protein
SLY430	-	Hypothetical Protein
SLY431	*ltrA*	Group II intron-encoded protein *ltrA*
SLY432	*ltrA*	Group II intron-encoded protein *ltrA*
SLY433	*dnaX*	DNA polymerase III gamma/tau subunit
SLY434	CHP	Conserved Hypothetical Protein
SLY435	CHP	Conserved Hypothetical Protein
SLY436	*pssA*	CDP-diacylglycerol--serine O-phosphatidyltransferase
SLY437	*psd2*	Phosphatidylserine decarboxylase proenzyme 2
SLY438	*pyrG*	CTP synthase
SLY439	CHP	Hypothetical Protein
SLY440	*dnaD*	DNA Replication Protein *DnaD*
SLY441	*rnc*	Ribonuclease III
SLY442	*plsX*	Fatty acid/phospholipid synthesis protein *plsX*
SLY443	-	Hypothetical Protein
SLY444	*norM*	Na+-Driven Multidrug Efflux Pump
SLY445	-	Hypothetical Protein
SLY446	*topA*	DNA topoisomerase I
SLY447	CHP	GTPase
SLY448	CHP	Conserved Hypothetical Protein
SLY449	*efp*	Elongation factor P
SLY450	*miaA*	tRNA delta(2)-isopentenylpyrophosphate transferase
SLY451	CHP	Hypothetical protein MG246
SLY452	CHP	Metal Dependent Phosphohydrolase
SLY453	CHP	Conserved Hypothetical Protein
SLY454	*yigN*	RmuC Family Protein
SLY455	CHP	Conserved Hypothetical Protein
SLY456	*gidA*	tRNA uridine 5-carboxymethylaminomethyl modification enzyme *gidA*
SLY457	*gidB*	Methyltransferase *gidB*
SLY458	*hflB*	Cell division protein *ftsH* homolog 4
SLY459	CHP	Conserved Hypothetical Protein
SLY460	*rpoD*	DNA-Directed RNA Polymerase Sigma Subunit
SLY461	CHP	Conserved Hypothetical Protein
SLY462	CHP	Phage-Associated Protein
SLY463	-	Hypothetical Protein
SLY464	*dnaC*	Replicative DNA helicase
SLY465	CHP	Conserved Hypothetical Protein associated with *tmk*
SLY466	*tmk*	Thymidylate kinase
SLY467	*tra5*	Putative transposase *tra5* for insertion sequence element IS150
SLY468	CHP	Conserved Hypothetical Protein
SLY469	*tuf*	Elongation factor Tu
SLY470	*fusA*	Elongation factor G
SLY471	*rpsG*	30S ribosomal protein S7
SLY472	*rpsL*	30S ribosomal protein S12
SLY473	*rpoC*	DNA-directed RNA polymerase beta' chain
SLY474	*rpoB*	DNA-directed RNA polymerase beta chain
SLY475	*rplL*	50S ribosomal protein L7/L12
SLY476	*rplJ*	50S ribosomal protein L10
SLY477	*rplA*	50S ribosomal protein L1
SLY478	*rplK*	50S ribosomal protein L11
SLY479	-	Hypothetical Protein
SLY480	*nusG*	Transcription antitermination protein *nusG*
SLY481	*secE*	Preprotein Translocase Subunit *SecE*
SLY482	*yacO*	TRNA/RRNA Methyltransferase
SLY483	-	Hypothetical Protein

ORF	Gene	Protein Function
SLY484	*exp7*	Probable cation-transporting ATPase exp7
SLY485	*pnpA*	Polyribonucleotide nucleotidyltransferase
SLY486	CHP	Conserved Hypothetical Protein (occurs within rRNA)
SLY487	-	Hypothetical Protein
SLY488	*oxaA2*	Membrane protein *oxaA2* precursor
SLY489	*rnpA*	Ribonuclease P protein component
SLY490	*rpmH*	50S ribosomal protein L34
SLY491	*proS*	Prolyl-tRNA synthetase
SLY492	-	Hypothetical Protein
SLY493	*pepP*	Xaa-Pro aminopeptidase
SLY494	*rpmG*	50S ribosomal protein L33
SLY495	CHP	Conserved Hypothetical Protein
SLY496	CHP	Conserved Hypothetical Protein
SLY497	-	Hypothetical Protein
SLY498	*hup*	DNA-binding protein HU
SLY499	*gpsA*	Glycerol-3-phosphate dehydrogenase
SLY500	-	Hypothetical Protein
SLY501	CHP	Conserved Hypothetical Protein
SLY502	-	Hypothetical Protein
SLY503	-	Hypothetical Protein
SLY504	*dnaB*	Replicative DNA helicase (fragment)
SLY505	-	Hypothetical Protein
SLY506	-	Hypothetical Protein
SLY507	*hpa*IM	Modification methylase *Hpa*I
SLY508	-	Hypothetical Protein
SLY509	*pdhA*	Pyruvate dehydrogenase E1 component, alpha subunit
SLY510	*pdhB*	Pyruvate dehydrogenase E1 component, beta subunit
SLY511	*pdhC*	Dihydrolipoyllysine-residue acetyltransferase component of pyruvate dehydrogenase complex
SLY512	*pdhD*	Dihydrolipoyl dehydrogenase
SLY513	*yabD*	Putative deoxyribonuclease *yabD*
SLY514	-	Hypothetical Protein
SLY515	-	Hypothetical Protein
SLY516	-	Hypothetical Protein
SLY517	CHP	Conserved Hypothetical Protein
SLY518	CHP	Conserved Hypothetical Protein
SLY519	*pcrA*	ATP-dependent DNA helicase *pcrA*
SLY520	*glnS*	Glutaminyl-tRNA synthetase
SLY521	-	Hypothetical Protein
SLY522	CHP	Conserved Hypothetical Protein
SLY523	-	Hypothetical Protein
SLY524	-	Hypothetical Protein
SLY525	-	Hypothetical Protein
SLY526	-	Hypothetical Protein
SLY527	-	Hypothetical Protein
SLY528	*uvrA*	UvrABC system protein A
SLY529	*tig*	Trigger factor
SLY530	*lon*	ATP-dependent protease La
SLY531	-	Hypothetical Protein
SLY532	*pmbA*	Protein *pmbA* homolog
SLY533	*tldD*	Protein *tldD*
SLY534	-	Hypothetical Protein
SLY535	-	Hypothetical Protein
SLY536	CHP	Conserved Hypothetical Protein
SLY537	*hit*	Protein hit
SLY538	CHP	Conserved Hypothetical Protein
SLY539	-	Hypothetical Protein
SLY540	-	Hypothetical Protein
SLY541	*rnhC*	Ribonuclease HIII
SLY542	*yhaM*	3'-5' exoribonuclease *yhaM*
SLY543	*cca*	CCA-adding enzyme
SLY544	*yaaJ*	Cytidine/Deoxycytidylate Deaminase Family Protein
SLY545	*tdk*	Thymidine kinase
SLY546	*rpmE*	50S ribosomal protein L31
SLY547	*rpmB*	50S ribosomal protein L28
SLY548	-	Hypothetical Protein
SLY549	-	Hypothetical Protein
SLY550	-	Hypothetical Protein

ORF	Gene	Protein Function
SLY551	CHP	Probable *Hsp20*-family chaperone
SLY552	*oppF*	Oligopeptide transport ATP-binding protein *oppF*
SLY553	-	Hypothetical Protein
SLY554	*tra5*	Putative transposase tra5 for insertion sequence element IS150
SLY555	CHP	Conserved Hypothetical Protein
SLY556	*hpa*IM	Modification methylase *Hpa*I (frag)
SLY557	-	Hypothetical Protein
SLY558	*rpoD*	DNA-Directed RNA Polymerase Sigma Subunit
SLY559	*ssb*	Single-strand binding protein (fragment)
SLY560	CHP	Conserved Hypothetical Protein
SLY561	CHP	Conserved Hypothetical Protein
SLY562	-	Hypothetical Protein
SLY563	-	Hypothetical Protein
SLY564	*yibP*	Hypothetical Protein *yibP*
SLY565	*rad50*	DNA double-strand break repair *rad50* ATPase
SLY566	*ftsH*	Cell division protein *ftsH* homolog
SLY567	*dnaC*	Replicative DNA helicase
SLY568	*dnaB*	Replicative DNA helicase
SLY569	CHP	Phage-Associated Protein
SLY570	*rpoD*	DNA-Directed RNA Polymerase Sigma Subunit
SLY571	*rpoD*	DNA-Directed RNA Polymerase Sigma Subunit
SLY572	CHP	Phage-Associated Protein
SLY573	CHP	Phage-Associated Protein
SLY574	*xor*IIM	Modification methylase *Xor*II
SLY575	CHP	Hypothetical Protein associated with *xor*IIM
SLY576	-	Phage-Associated Protein
SLY577	-	Hypothetical Protein
SLY578	*dnaC*	Replicative DNA helicase
SLY579	CHP	Conserved Hypothetical Protein associated with *tmk*
SLY580	*tmk*	Thymidylate kinase
SLY581	-	Hypothetical Protein
SLY582	llaDCHIA	Modification methylase LlaDCHIA
SLY583	CHP	Phage-Associated Protein
SLY584	CHP	Phage-Associated Protein
SLY585	-	Hypothetical Protein
SLY586	*ihfA*	Hypothetical Protein *ihfA*
SLY587	CHP	Conserved Hypothetical Protein (methylase)
SLY588	-	Hypothetical Protein
SLY589	-	Hypothetical Protein
SLY590	CHP	Conserved Hypothetical Protein
SLY591	CHP	Conserved Hypothetical Protein
SLY592	*hflB*	Putative cell division cycle ATPase
SLY593	CHP	Polyprotein -Paragroups CHP041 and CHP152
SLY594	CHP	Conserved Hypothetical Protein
SLY595	CHP	Conserved Hypothetical Protein
SLY596	*yibP*	Hypothetical Protein *yibP* (fragment)
SLY597	CHP	Conserved Hypothetical Protein
SLY598	CHP	Phage-Associated Protein
SLY599	-	Hypothetical Protein
SLY600	-	Hypothetical Protein
SLY601	CHP	Conserved Hypothetical Protein
SLY602	*tra5*	Putative transposase *tra5* for insertion sequence element IS150
SLY603	*tra5*	Putative transposase *tra5* for insertion sequence element IS150
SLY604	-	Hypothetical Protein
SLY605	*tra5*	Putative transposase *tra5* for insertion sequence element IS150
SLY606	CHP	Conserved Hypothetical Protein
SLY607	-	Phage-Associated Protein
SLY608	-	Hypothetical Protein
SLY609	-	Hypothetical Protein
SLY610	-	Hypothetical Protein
SLY611	-	Hypothetical Protein
SLY612	*yibP*	Hypothetical Protein *yibP*

ORF	Gene	Protein Function
SLY613	*rad50*	DNA double-strand break repair *rad50* ATPase
SLY614	*ftsH*	Cell division protein *ftsH* homolog
SLY615	CHP	Conserved Hypothetical Protein
SLY616	-	Hypothetical Protein
SLY617	-	Hypothetical Protein
SLY618	CHP	Conserved Hypothetical Protein
SLY619	-	Hypothetical Protein
SLY620	-	Hypothetical Protein
SLY621	CHP	Conserved Hypothetical Protein (methylase)
SLY622	-	Hypothetical Protein
SLY623	CHP	Conserved Hypothetical Protein
SLY624	CHP	Conserved Hypothetical Protein
SLY625	*ihfA*	Hypothetical Protein *ihfA*
SLY626	CHP	Conserved Hypothetical Protein
SLY627	-	Hypothetical Protein
SLY628	CHP	Phage-Associated Protein
SLY629	*tmk*	Thymidylate kinase
SLY630	CHP	Conserved Hypothetical Protein associated with *tmk*
SLY631	*dnaC*	Replicative DNA helicase
SLY632	-	Hypothetical Protein
SLY633	CHP	Phage-Associated Protein
SLY634	*xor*IIM	Modification methylase *Xor*II
SLY635	-	Hypothetical Protein
SLY636	-	Phage-Associated Protein
SLY637	-	Hypothetical Protein
SLY638	*rpoD*	DNA-Directed RNA Polymerase Sigma Subunit
SLY639	CHP	Conserved Hypothetical Protein
SLY640	*tra5*	Putative transposase tra5 for insertion sequence element IS150
SLY641	*rpoD*	DNA-Directed RNA Polymerase Sigma Subunit
SLY642	*tmk*	Thymidylate kinase
SLY643	CHP	Conserved Hypothetical Protein associated with *tmk*
SLY644	*dnaC*	Replicative DNA helicase
SLY645	*yibP*	Hypothetical Protein yibP
SLY646	*yibP*	Hypothetical Protein yibP
SLY647	CHP	Conserved Hypothetical Protein
SLY648	-	Hypothetical Protein
SLY649	*hmw2*	Cytadherence high molecular weight protein 2
SLY650	-	Hypothetical Protein
SLY651	-	Hypothetical Protein
SLY652	*oppF*	Oligopeptide transport ATP-binding protein *oppF*
SLY653	*oppD*	Oligopeptide transport ATP-binding protein *oppD*
SLY654	-	Hypothetical Protein
SLY655	*appA*	Oligopeptide-binding protein *appA* precursor
SLY656	*dppA*	Periplasmic dipeptide transport protein precursor
SLY657	-	Hypothetical Protein
SLY658	*oppB*	Oligopeptide transport system permease protein *oppB*
SLY659	*yliD*	Oligopeptide ABC Transporter Permease Protein
SLY660	*potA*	Spermidine/putrescine import ATP-binding protein *potA* (fragment)
SLY661	-	Hypothetical Protein
SLY662	*groS*	10 kDa chaperonin
SLY663	*groL*	60 kDa chaperonin
SLY664	*amp*	Antigenic membrane protein precursor
SLY665	*nadE*	Glutamine-dependent NAD(+) synthetase
SLY666	-	Hypothetical Protein
SLY667	-	Hypothetical Protein
SLY668	CHP	Conserved Hypothetical Protein
SLY669	*trmU*	Probable tRNA
SLY670	-	Hypothetical Protein
SLY671	*relA*	GTP pyrophosphokinase
SLY672	-	Hypothetical Protein
SLY673	-	Hypothetical Protein
SLY674	*citP*	Citrate-sodium symport
SLY675	*ytsJ*	Probable NAD-dependent malic enzyme 4
SLY676	-	Hypothetical Protein
SLY677	*ligA*	DNA ligase
SLY678	CHP	Hypothetical protein MG103 homolog
SLY679	-	Hypothetical Protein
SLY680	-	Hypothetical Protein
SLY681	-	Hypothetical Protein
SLY682	-	Hypothetical Protein
SLY683	*rpoD*	DNA-Directed RNA Polymerase Sigma Subunit
SLY684	-	Hypothetical Protein
SLY685	CHP	Phage-Associated Protein
SLY686	-	Hypothetical Protein
SLY687	*xor*IIM	Modification methylase *Xor*II
SLY688	CHP	Phage-Associated Protein
SLY689	-	Hypothetical Protein
SLY690	*dnaC*	Replicative DNA helicase
SLY691	CHP	Conserved Hypothetical Protein associated with *tmk*
SLY692	*tmk*	Thymidylate kinase
SLY693	llaDCHIA	Modification methylase LlaDCHIA
SLY694	CHP	Phage-Associated Protein
SLY695	CHP	Phage-Associated Protein
SLY696	CHP	Conserved Hypothetical Protein
SLY697	*ihfA*	Hypothetical Protein *ihfA*
SLY698	-	Hypothetical Protein
SLY699	-	Hypothetical Protein
SLY700	CHP	Conserved Hypothetical Protein (methylase)
SLY701	-	Hypothetical Protein
SLY702	-	Hypothetical Protein
SLY703	CHP	Conserved Hypothetical Protein
SLY704	CHP	Conserved Hypothetical Protein
SLY705	CHP	Conserved Hypothetical Protein
SLY706	CHP	Hypothetical Protein possibly *ftsH*-associated
SLY707	*ftsH*	Cell division protein *ftsH* homolog
SLY708	*rad50*	DNA double-strand break repair rad50 ATPase
SLY709	*yibP*	Hypothetical Protein *yibP*
SLY710	CHP	Phage-Associated Protein
SLY711	-	Hypothetical Protein
SLY712	*tra5*	Putative transposase *tra5* for insertion sequence element IS150
SLY713	*tra5*	Putative transposase *tra5* for insertion sequence element IS150
SLY714	*dnaC*	Replicative DNA helicase
SLY715	-	Hypothetical Protein
SLY716	*yibP*	Hypothetical Protein *yibP*
SLY717	*rad50*	DNA double-strand break repair *rad50* ATPase
SLY718	*ftsH*	Cell division protein *ftsH* homolog
SLY719	-	Hypothetical Protein
SLY720	CHP	Hypothetical Protein possibly *ftsH*-associated
SLY721	-	Hypothetical Protein
SLY722	-	Hypothetical Protein
SLY723	CHP	Conserved Hypothetical Protein
SLY724	-	Hypothetical Protein
SLY725	-	Hypothetical Protein
SLY726	CHP	Conserved Hypothetical Protein (methylase)
SLY727	*hupB*	Hypothetical Protein *hupB*
SLY728	-	Hypothetical Protein
SLY729	llaDCHIA	Modification methylase LlaDCHIA
SLY730	*tmk*	Thymidylate kinase
SLY731	CHP	Conserved Hypothetical Protein associated with *tmk*
SLY732	*dnaC*	Replicative DNA helicase
SLY733	-	Hypothetical Protein

ORF	Gene	Protein Function
SLY734	*rpoD*	DNA-Directed RNA Polymerase Sigma Subunit
SLY735	*glyA*	Serine hydroxymethyltransferase
SLY736	*cbiQ*	ABC Transporter Permease Protein
SLY737	*cbiO2*	Cobalt import ATP-binding protein *cbiO2*
SLY738	*cbiO2*	Putative cobalt import ATP-binding protein *cbiO2*
SLY739	*yjbG*	Oligoendopeptidase F homolog
SLY740	-	Hypothetical Protein
SLY741	CHP	Conserved Hypothetical Protein
SLY742	CHP	Conserved Hypothetical Protein
SLY743	-	Hypothetical Protein
SLY744	-	Hypothetical Protein
SLY745	-	Hypothetical Protein
SLY746	*ltrA*	Group II intron-encoded protein *ltrA* [Includes: Reverse-transcriptase
SLY747	*gyrB*	DNA gyrase subunit B
SLY748	*gyrA*	DNA gyrase subunit A
SLY749	*yjeE*	Hypothetical UPF0079 protein *yjeE*
SLY750	*yeaZ*	Probable M22 peptidase homolog HI0388
SLY751	-	Hypothetical Protein
SLY752	-	Hypothetical Protein
SLY753	-	Hypothetical Protein
SLY754	-	Hypothetical Protein
SLY755	CHP	Conserved Hypothetical Protein
SLY756	-	Hypothetical Protein
SLY757	*ycjM*	Sucrose phosphorylase
SLY758	*lepA*	GTP-binding protein *lepA*
SLY759	-	Hypothetical Protein
SLY760	-	Hypothetical Protein
SLY761	-	Hypothetical Protein
SLY762	-	Hypothetical Protein
SLY763	*tra5*	Putative transposase *tra5* for insertion sequence element IS150
SLY764	CHP	Conserved Hypothetical Protein
SLY765	CHP	Hypothetical protein -Paragroup CHP041
SLY766	*tra5*	Putative transposase *tra5* for insertion sequence element IS150
SLY767	-	Hypothetical Protein
SLY768	-	Hypothetical Protein
SLY769	*yibP*	Hypothetical Protein *yibP*
SLY770	*rad50*	DNA double-strand break repair *rad50* ATPase
SLY771	*ftsH*	Cell division protein ftsH homolog
SLY772	-	Hypothetical Protein
SLY773	CHP	Hypothetical Protein possibly ftsH-associated
SLY774	-	Hypothetical Protein
SLY775	-	Hypothetical Protein
SLY776	CHP	Conserved Hypothetical Protein
SLY777	-	Hypothetical Protein
SLY778	-	Hypothetical Protein
SLY779	CHP	Conserved Hypothetical Protein (methylase)
SLY780	*hupB*	Hypothetical Protein *hupB*
SLY781	CHP	Conserved Hypothetical Protein
SLY782	CHP	Phage-Associated Protein
SLY783	*tmk*	Thymidylate kinase
SLY784	CHP	Conserved Hypothetical Protein associated with *tmk*
SLY785	*dnaC*	Replicative DNA helicase
SLY786	-	Hypothetical Protein
SLY787	*rpoD*	DNA-Directed RNA Polymerase Sigma Subunit
SLY788	*infC*	Translation initiation factor IF-3
SLY789	*rpmI*	50S ribosomal protein L35
SLY790	*rplT*	50S ribosomal protein L20
SLY791	CHP	Conserved Hypothetical Protein
SLY792	*acpP*	Acyl carrier protein
SLY793	*ltrA*	Retron-Type Reverse Transcriptase
SLY794	*ltrA*	Group II intron-encoded protein *ltrA* [Includes: Reverse-transcriptase
SLY795	-	Hypothetical Protein

ORF	Gene	Protein Function
SLY796	*leuS*	Leucyl-tRNA synthetase
SLY797	*gmk*	Guanylate kinase
SLY798	*rpoZ*	DNA-Directed RNA Polymerase Subunit K/Omega
SLY799	*mraW*	S-adenosyl-methyltransferase *mraW*
SLY800	*tra5*	Putative transposase *tra5* for insertion sequence element IS150
SLY801	CHP	Conserved Hypothetical Protein
SLY802	*yeeO*	Na+ Driven Multidrug Efflux Pump
SLY803	CHP	Conserved Hypothetical Protein
SLY804	-	Hypothetical Protein
SLY805	CHP	Conserved Hypothetical Protein
SLY806	*nrdF*	Ribonucleoside-diphosphate reductase beta subunit
SLY807	*mscL*	Large-conductance mechanosensitive channel
SLY808	-	Hypothetical Protein
SLY809	*yfiB*	ABC Transporter
SLY810	*msbA*	ABC Transporter
SLY811	*tra5*	Putative transposase *tra5* for insertion sequence element IS150
SLY812	-	Hypothetical Protein
SLY813	-	Hypothetical Protein
SLY814	*ltrA*	Group II intron-encoded protein *ltrA* [Includes: Reverse-transcriptase
SLY815	-	Hypothetical Protein
SLY816	CHP	Hypothetical protein -Paragroup CHP041
SLY817	CHP	Hypothetical protein -Paragroup CHP158
SLY818	-	Hypothetical Protein
SLY819	*hflB*	Putative cell division cycle ATPase
SLY820	-	Hypothetical Protein
SLY821	CHP	Conserved Hypothetical Protein
SLY822	-	Hypothetical Protein
SLY823	-	Hypothetical Protein
SLY824	CHP	Conserved Hypothetical Protein (methylase)
SLY825	*hupB*	Hypothetical Protein *hupB*
SLY826	-	Hypothetical Protein
SLY827	-	Hypothetical Protein
SLY828	-	Hypothetical Protein
SLY829	llaDCHIA	Modification methylase LlaDCHIA
SLY830	CHP	Conserved Hypothetical Protein
SLY831	-	Hypothetical Protein
SLY832	*tmk*	Thymidylate kinase
SLY833	CHP	Conserved Hypothetical Protein associated with *tmk*
SLY834	*dnaC*	Replicative DNA helicase
SLY835	-	Hypothetical Protein
SLY836	-	Hypothetical Protein
SLY837	-	Hypothetical Protein
SLY838	-	Hypothetical Protein
SLY839	*yplQ*	Hemolysin III homolog
SLY840	*ackA*	Acetate kinase
SLY841	-	Hypothetical Protein
SLY842	-	Hypothetical Protein
SLY843	*priA*	Primosomal protein N'
SLY844	-	Hypothetical Protein
SLY845	-	Hypothetical Protein
SLY846	CHP	Conserved Hypothetical Protein
SLY847	CHP	Conserved Hypothetical Protein
SLY848	-	Hypothetical Protein
SLY849	*tra5*	Putative transposase *tra5* for insertion sequence element IS150
SLY850	*tra5*	Putative transposase *tra5* for insertion sequence element IS150
SLY851	llaDCHIA	Modification methylase LlaDCHIA
SLY852	*tmk*	Thymidylate kinase
SLY853	CHP	Conserved Hypothetical Protein associated with *tmk*
SLY854	*dnaC*	Replicative DNA helicase
SLY855	-	Hypothetical Protein
SLY856	CHP	Phage-Associated Protein
SLY857	-	Hypothetical Protein

ORF	Gene	Protein Function
SLY858	*rpoD*	DNA-Directed RNA Polymerase Sigma Subunit
SLY859	*metG*	Methionyl-tRNA synthetase
SLY860	-	Hypothetical Protein
SLY861	*pepA*	Probable cytosol aminopeptidase
SLY862	-	Hypothetical Protein
SLY863	-	Hypothetical Protein
SLY864	-	Hypothetical Protein
SLY865	-	Hypothetical Protein
SLY866	*potD*	Spermidine/putrescine-binding periplasmic protein precursor
SLY867	*potC*	Spermidine/putrescine transport system permease protein *potC*
SLY868	*potB*	Spermidine/putrescine transport system permease protein *potB*
SLY869	*potA*	Spermidine/putrescine import ATP-binding protein *potA*
SLY870	-	Hypothetical Protein
SLY871	*ftsH*	Cell division protein *ftsH* homolog
SLY872	-	Hypothetical Protein
SLY873	-	Hypothetical Protein
SLY874	CHP	Conserved Hypothetical Protein
SLY875	*cbiQ*	ABC-type cobalt transporter, permease component
SLY876	*cbiO1*	Putative cobalt import ATP-binding protein *cbiO1*
SLY877	CHP	Integral Membrane Protein
SLY878	-	Hypothetical Protein
SLY879	-	Hypothetical Protein
SLY880	-	Hypothetical Protein
SLY881	-	Hypothetical Protein
SLY882	*valS*	Valyl-tRNA synthetase
SLY883	*engB*	Probable GTP-binding protein *engB*
SLY884	-	Hypothetical Protein
SLY885	*yqeN*	Hypothetical protein yqeN
SLY886	*yciO*	Translation Factor
SLY887	*prfA*	Peptide chain release factor 1
SLY888	*dnaC*	Replicative DNA helicase
SLY889	*rplI*	50S Ribosomal Protein L9
SLY890	*rpsR*	30S ribosomal protein S18
SLY891	*ssb1*	Single-strand binding protein 1
SLY892	*rpsF*	30S ribosomal protein S6
SLY893	-	Hypothetical Protein
SLY894	-	Hypothetical Protein
SLY895	-	Hypothetical Protein
SLY896	-	Hypothetical Protein
SLY897	-	Hypothetical Protein
SLY898	*tra5*	Putative transposase *tra5* for insertion sequence element IS150
SLY899	-	Hypothetical Protein
SLY900	*srmB*	Probable ATP-dependent RNA helicase srmB
SLY901	-	Hypothetical Protein
SLY902	-	Hypothetical Protein
SLY903	-	Hypothetical Protein
SLY904	-	Hypothetical Protein
SLY905	-	Hypothetical Protein
SLY906	-	Hypothetical Protein
SLY907	-	Hypothetical Protein
SLY908	CHP	Conserved Hypothetical Protein
SLY909	-	Hypothetical Protein
SLY910	-	Hypothetical Protein
SLY911	*pfkA*	6-phosphofructokinase
SLY912	CHP	Conserved Hypothetical Protein
SLY913	*norM*	Na+-Driven Multidrug Efflux Pump
SLY914	*pgi*	Glucose-6-phosphate isomerase
SLY915	-	Hypothetical Protein
SLY916	*yraL*	Tetrapyrrole Methylase Family Protein
SLY917	*holB*	DNA polymerase III delta' subunit
SLY918	*tmk*	Thymidylate kinase (tmk-b)
SLY919	*truA*	tRNA pseudouridine synthase A
SLY920	*norM*	Na+-Driven Multidrug Efflux Pump
SLY921	-	Hypothetical Protein
SLY922	*tra5*	Putative transposase *tra5* for insertion sequence element IS150
SLY923	-	Hypothetical Protein
SLY924	-	Hypothetical Protein
SLY925	-	Hypothetical Protein
SLY926	CHP	Phage-Associated Protein
SLY927	*tra5*	Putative transposase *tra5* for insertion sequence element IS150
SLY928	CHP	Conserved Hypothetical Protein
SLY929	CHP	Hypothetical protein -Paragroup CHP041
SLY930	CHP	Hypothetical protein -Paragroup CHP152
SLY931	*hflB*	Putative cell division cycle ATPase
SLY932	CHP	Conserved Hypothetical Protein (methylase)
SLY933	CHP	Phage-Associated Protein
SLY934	-	Hypothetical Protein
SLY935	-	Hypothetical Protein
SLY936	CHP	Conserved Hypothetical Protein
SLY937	-	Hypothetical Protein
SLY938	-	Hypothetical Protein
SLY939	*ihfA*	Hypothetical Protein *ihfA*
SLY940	CHP	Conserved Hypothetical Protein
SLY941	-	Hypothetical Protein
SLY942	-	Hypothetical Protein
SLY943	-	Hypothetical Protein
SLY944	CHP	Phage-Associated Protein
SLY945	-	Hypothetical Protein
SLY946	*tmk*	Thymidylate kinase
SLY947	CHP	Conserved Hypothetical Protein associated with *tmk*
SLY948	*dnaB*	Replicative DNA helicase (fragment)
SLY949	*rpoD*	DNA-Directed RNA Polymerase Sigma Subunit
SLY950	CHP	Conserved Hypothetical Protein
SLY951	CHP	Phage-Associated Protein
SLY952	-	Hypothetical Protein
SLY953	*xor*IIM	Modification methylase *Xor*II
SLY954	CHP	Phage-Associated Protein
SLY955	-	Hypothetical Protein
SLY956	*dnaC*	Replicative DNA helicase
SLY957	CHP	Conserved Hypothetical Protein associated with tmk
SLY958	*tmk*	Thymidylate kinase
SLY959	llaDCHIA	Modification methylase LlaDCHIA
SLY960	CHP	Phage-Associated Protein
SLY961	CHP	Phage-Associated Protein
SLY962	CHP	Conserved Hypothetical Protein
SLY963	*ihfA*	Hypothetical Protein ihfA
SLY964	-	Hypothetical Protein
SLY965	-	Hypothetical Protein
SLY966	CHP	Conserved Hypothetical Protein (methylase)
SLY967	-	Hypothetical Protein
SLY968	-	Hypothetical Protein
SLY969	CHP	Conserved Hypothetical Protein
SLY970	-	Hypothetical Protein
SLY971	-	Hypothetical Protein
SLY972	CHP	Hypothetical Protein possibly *ftsH*-associated
SLY973	*ftsH*	Cell division protein *ftsH* homolog
SLY974	*rad50*	DNA double-strand break repair rad50 ATPase
SLY975	*yibP*	Hypothetical Protein *yibP*
SLY976	CHP	Conserved Hypothetical Protein
SLY977	CHP	Phage-Associated Protein
SLY978	-	Hypothetical Protein
SLY979	-	Hypothetical Protein
SLY980	*rpoD*	DNA-Directed RNA Polymerase Sigma Subunit
SLY981	CHP	Conserved Hypothetical Protein
SLY982	*dnaC*	Replicative DNA helicase
SLY983	CHP	Conserved Hypothetical Protein associated with tmk
SLY984	*tmk*	Thymidylate kinase
SLY985	llaDCHIA	Modification methylase LlaDCHIA

ORF	Gene	Protein Function
SLY986	-	Hypothetical Protein
SLY987	-	Hypothetical Protein
SLY988	*ihfA*	Hypothetical Protein ihfA
SLY989	CHP	Conserved Hypothetical Protein (methylase)
SLY990	-	Hypothetical Protein
SLY991	CHP	Phage-Associated Protein
SLY992	CHP	Phage-Associated Protein
SLY993	CHP	Phage-Associated Protein
SLY994	-	Hypothetical Protein
SLY995	CHP	Conserved Hypothetical Protein
SLY996	CHP	Conserved Hypothetical Protein
SLY997	CHP	Conserved Hypothetical Protein
SLY998	-	Hypothetical Protein
SLY999	*vat*	VCP-like ATPase
SLY1000	CHP	Hypothetical protein -Paragroup CHP152
SLY1001	CHP	Hypothetical protein -Paragroup CHP041
SLY1002	-	Hypothetical Protein
SLY1003	-	Hypothetical Protein
SLY1004	*tra5*	Putative transposase *tra5* for insertion sequence element IS150
SLY1005	-	Hypothetical Protein
SLY1006	*tra5*	Putative transposase *tra5* for insertion sequence element IS150
SLY1007	CHP	Conserved Hypothetical Protein MSC
SLY1008	-	Hypothetical Protein
SLY1009	*yqiZ*	Probable amino-acid ABC transporter ATP-binding protein yqiZ
SLY1010	*mutT*	MutT/Nudix Family Protein
SLY1011	-	Hypothetical Protein
SLY1012	CHP	Conserved Hypothetical Protein (methylase)
SLY1013	*pacL*	Cation-transporting ATPase pacL
SLY1014	*engA*	GTP-binding protein engA
SLY1015	*cmk*	Cytidylate kinase
SLY1016	*rluB*	Ribosomal large subunit pseudouridine synthase B
SLY1017	*truB*	tRNA pseudouridine synthase B
SLY1018	*trxA*	Thioredoxin
SLY1019	CHP	Conserved Hypothetical Protein
SLY1020	*rpoD*	DNA-Directed RNA Polymerase Sigma Subunit
SLY1021	CHP	Conserved Hypothetical Protein
SLY1022	*dnaC*	Replicative DNA helicase
SLY1023	CHP	Conserved Hypothetical Protein associated with tmk
SLY1024	*tmk*	Thymidylate kinase
SLY1025	-	Hypothetical Protein
SLY1026	llaDCHIA	Modification methylase LlaDCHIA
SLY1027	*rpoD*	DNA-Directed RNA Polymerase Sigma Subunit
SLY1028	*ssb*	Single-strand binding protein
SLY1029	-	Hypothetical Protein
SLY1030	CHP	Conserved Hypothetical Protein
SLY1031	*ssb*	Single-strand binding protein
SLY1032	-	Hypothetical Protein
SLY1033	-	Hypothetical Protein
SLY1034	-	Hypothetical Protein
SLY1035	-	Hypothetical Protein
SLY1036	-	Hypothetical Protein
SLY1037	-	Phage-Associated Protein
SLY1038	-	Hypothetical Protein
SLY1039	-	Hypothetical Protein
SLY1040	*xor*IIM	Modification methylase *Xor*II (fragment)
SLY1041	*xor*IIM	Modification methylase *Xor*II (fragment)
SLY1042	*tra5*	Putative transposase tra5 for insertion sequence element IS150
SLY1043	*tra5*	Putative transposase tra5 for insertion sequence element IS150
SLY1044	-	Hypothetical Protein
SLY1045	-	Hypothetical Protein

ORF	Gene	Protein Function
SLY1046	*recA*	Protein *recA* (fragment)
SLY1047	*recA*	Protein *recA* (fragment)
SLY1048	*recA*	Protein *recA* (fragment)
SLY1049	CHP	Conserved Hypothetical Protein
SLY1050	-	Hypothetical Protein
SLY1051	-	Hypothetical Protein
SLY1052	*pgsA*	CDP-diacylglycerol--glycerol-3-phosphate 3-phosphatidyltransferase
SLY1053	-	Hypothetical Protein
SLY1054	-	Hypothetical Protein
SLY1055	-	Hypothetical Protein
SLY1056	-	Hypothetical Protein
SLY1057	-	Hypothetical Protein
SLY1058	CHP	Conserved Hypothetical Protein
SLY1059	*Rock2*	Rho-associated protein kinase 2
SLY1060	CHP	DAK2 Domain Protein
SLY1061	*csp*	Cold shock protein 1
SLY1062	CHP	Conserved Hypothetical Protein
SLY1063	*dnaI*	Primosomal protein *dnaI*
SLY1064	*dnaB*	Replication initiation and membrane attachment protein
SLY1065	*mutM*	Formamidopyrimidine-DNA glycosylase
SLY1066	-	Hypothetical Protein
SLY1067	*polA*	DNA polymerase I
SLY1068	*ffh*	ffh frag
SLY1069	*ffh*	ffh frag
SLY1070	*ffh*	Signal recognition particle protein
SLY1071	*ftsY*	Cell division protein *ftsY* homolog
SLY1072	-	Hypothetical Protein
SLY1073	*rpoD*	DNA-Directed RNA Polymerase Sigma Subunit
SLY1074	-	Hypothetical Protein
SLY1075	CHP	Phage-Associated Protein
SLY1076	*dnaC*	Replicative DNA helicase
SLY1077	CHP	Conserved Hypothetical Protein associated with *tmk*
SLY1078	*tmk*	Thymidylate kinase
SLY1079	llaDCHIA	Modification methylase LlaDCHIA
SLY1080	CHP	Phage-Associated Protein
SLY1081	*dnaC*	Replicative DNA helicase
SLY1082	CHP	Conserved Hypothetical Protein associated with tmk
SLY1083	*tmk*	Thymidylate kinase
SLY1084	*dnaC*	Replicative DNA helicase
SLY1085	CHP	Conserved Hypothetical Protein associated with tmk
SLY1086	*tmk*	Thymidylate kinase
SLY1087	-	Hypothetical Protein
SLY1088	llaDCHIA	Modification methylase LlaDCHIA
SLY1089	-	Hypothetical Protein
SLY1090	*hupB*	Hypothetical Protein hupB / ihfA
SLY1091	-	Hypothetical Protein
SLY1092	CHP	Conserved Hypothetical Protein
SLY1093	CHP	Conserved Hypothetical Protein
SLY1094	-	Hypothetical Protein
SLY1095	-	Hypothetical Protein
SLY1096	-	Hypothetical Protein possibly ftsH-associated
SLY1097	-	Hypothetical Protein
SLY1098	*ftsH*	Cell division protein ftsH homolog
SLY1099	*rad50*	DNA double-strand break repair rad50 ATPase
SLY1100	*yibP*	Hypothetical Protein yibP
SLY1101	-	Hypothetical Protein
SLY1102	-	Hypothetical Protein
SLY1103	-	Hypothetical Protein
SLY1104	*tra5*	Transposase
SLY1105	*tra5*	Putative transposase *tra5* for insertion sequence element IS150
SLY1106	CHP	Conserved Hypothetical Protein (occurs in rRNA region)
SLY1107	-	Hypothetical Protein
SLY1108	-	Hypothetical Protein
SLY1109	-	Hypothetical Protein

ORF	Gene	Protein Function
SLY1110	-	Hypothetical Protein
SLY1111	-	Hypothetical Protein
SLY1112	*fic*	Fic Family Protein
SLY1113	-	Hypothetical Protein
SLY1114	-	Hypothetical Protein
SLY1115	-	Hypothetical Protein
SLY1116	-	Hypothetical Protein
SLY1117	-	Hypothetical Protein
SLY1118	-	Hypothetical Protein
SLY1119	-	Hypothetical Protein
SLY1120	-	Hypothetical Protein
SLY1121	*nusB*	N utilization substance protein B homolog
SLY1122	*sodA*	Superoxide dismutase [Mn]
SLY1123	*nox*	NADH oxidase
SLY1124	-	Hypothetical Protein
SLY1125	*acpS*	Holo-[acyl-carrier-protein] synthase
SLY1126	-	Hypothetical Protein
