# Supplementary material for: Comparison of the complete genome sequence of two closely related isolates of ‘Candidatus Phytoplasma australiense’ reveals genome plasticity
Source: BMC Genomics. 2013 Aug 2;14:529. doi: 10.1186/1471-2164-14-529 (PMC3750655; doi:10.1186/1471-2164-14-529)

Additional file 2

Figure S1. Graphical representation of size and distribution of areas consisting of Potential Mobile Units (PMUs) in the SLY genome.

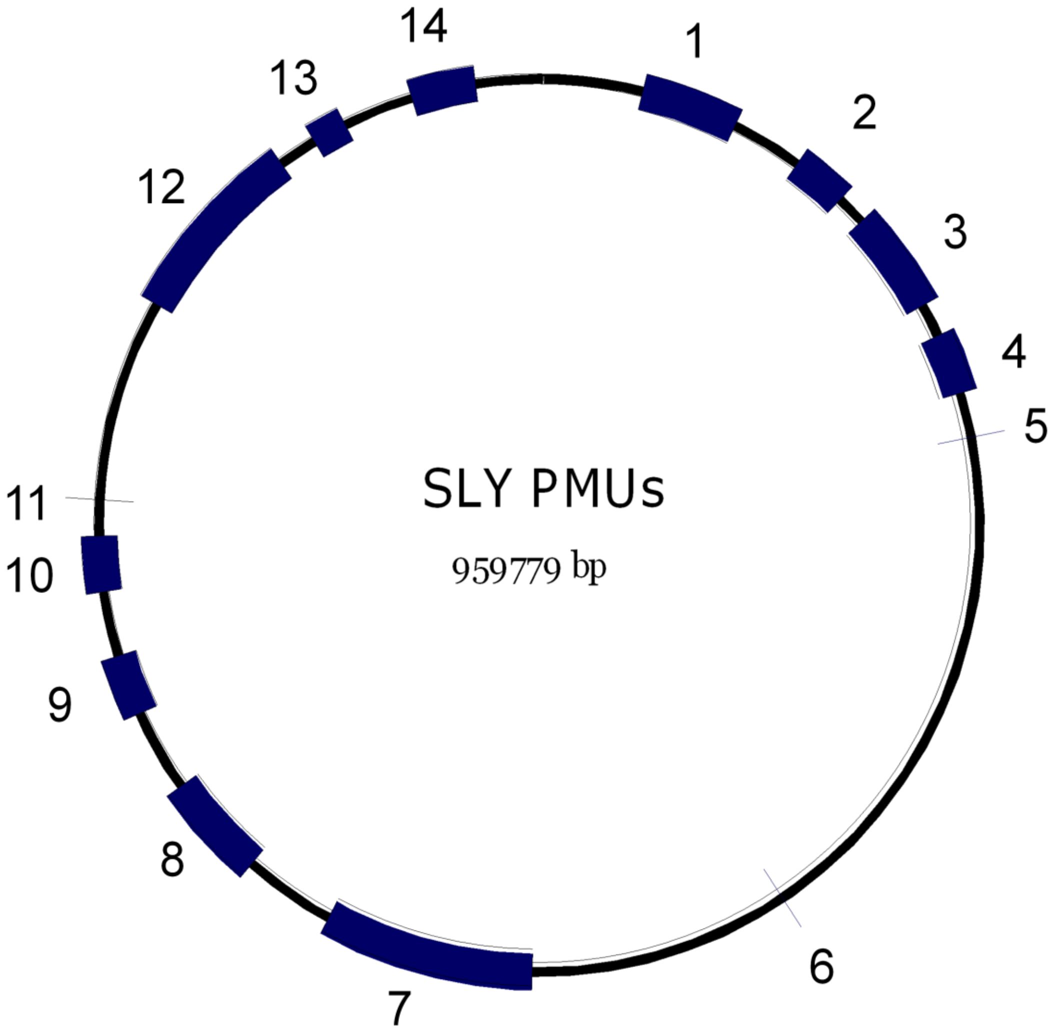

Supplement: Additional file 2 — Potential Mobile Units (PMUs) in the SLY genome. Graphical representation of size and distribution of areas consisting of Potential Mobile Units (PMUs) in the SLY genome. [file 1471-2164-14-529-S2.pdf]
