# Supplementary material for: Comparison of the complete genome sequence of two closely related isolates of ‘Candidatus Phytoplasma australiense’ reveals genome plasticity
Source: BMC Genomics. 2013 Aug 2;14:529. doi: 10.1186/1471-2164-14-529 (PMC3750655; doi:10.1186/1471-2164-14-529)
Supplement: Additional file 3 — Potential Mobile Units (PMUs) of PAa. Size of PAa Potential Mobile Units (PMUs) as determined using Open Reading Frame (ORF) boundaries. Boundaries were determined by the association of ORFs with PMUs, including hypothetical proteins (HP) and conserved hypothetical proteins (CHP). [file 1471-2164-14-529-S3.pdf]

Additional file 3

Table S2. Size of PAa Potential Mobile Units (PMUs) as determined using Open Reading Frame (ORF) boundaries. Boundaries were determined by the association of ORFs with PMUs, including hypothetical proteins (HP) and conserved hypothetical proteins (CHP).

PAa PMU#	ORF start		ORF finish		Nucl start	Nucl. finish	Size (bp)
1	PA0027	HP	PA0055	*ftsH*	34337	55910	21573
2	PA0062	HP	PA0081	HP	68558	88112	19554
3	PA0179	HP	PA0212	Retron-type reverse transcriptase, fragment 2	217613	249777	32164
4	PA0225	Replicative DNA helicase, prophage	PA0265	*tra5* (Putative phage integrase, fragment)	265947	294714	28767
5	PA0275	*tra5* (Putative phage integrase, fragment)	PA0344	CHP	305272	355675	50403
6	PA0351	Putative phage integrase	PA0448	Putative endonuclease IV, fragment	366099	444633	78534
7	PA0457	HP	PA0480	HP	457682	475649	17967
8	PA0646	ATP-dependent Zn protease, fragment	PA0659	CHP	646382	656742	10360
9	PA0722	Putative methylase	PA0751	Putative phage integrase, fragment	738721	761578	22857
10	PA0785	HP	PA0815	DNA-directed RNA polymerase specialized sigma subunit (*rpoD*)	806241	834650	28409
