# Supplementary material for: Comparison of the complete genome sequence of two closely related isolates of ‘Candidatus Phytoplasma australiense’ reveals genome plasticity
Source: BMC Genomics. 2013 Aug 2;14:529. doi: 10.1186/1471-2164-14-529 (PMC3750655; doi:10.1186/1471-2164-14-529)
Supplement: Additional file 4 — Comparison of a selection of “housekeeping” genes of PAa and SLY and OY-M and AY-WB. DNA comparison of a selection of “housekeeping” genes of two ‘Candidatus Phytoplasma australiense’ isolates PAa and SLY (A) and two ’Ca. Phytoplasma asteris’ isolates OY-M and AY-WB (B). Most genes were present in each genome as a single copy. Two copies of mtgA were present in OY-M and AY-WB, and those with comparable syntenic positions were compared. [file 1471-2164-14-529-S4.pdf]

Additional file 4

Table S3. DNA comparison of a selection of "housekeeping" genes of two '*Ca*. Phytoplasma australiense' isolates PAa and SLY (A) and two '*Ca*. Phytoplasma asteris' isolates OY-M and AY-WB (B). Most genes were present in each genome as a single copy. Two copies of *mtgA* were present in OY-M and AY-WB, and those with comparable syntenic positions were compared.

A

Gene	PAa	SLY	% similarity (DNA)
tuf	PA0660	SLY469	100
eno	PA0059	SLY079	100
rplA	PA0668	SLY477	100
rpoB	PA0665	SLY474	100
dnaA	PA0001	SLY001	100
tmk-b	PA0169	SLY918	100
secA	PA0551	SLY349	100
secE	PA0671	SLY481	100
secY	PA0567	SLY368	100
potA	PA0135	SLY869	100
potB	PA0134	SLY868	100
potC	PA0133	SLY867	100
potD	PA0132	SLY866	100
oppB	PA0757	SLY658	100
oppD	PA0754	SLY653	99
oppE/F	PA0718	SLY552	99
oppF	PA0753	SLY652	100
amp	PA0762	SLY664	100
appA	PA0755	SLY655	100
dppA	PA0756	SLY656	100
mgtA	PA0089	SLY098	100
malE	PA0022	SLY027	100<br>99 (with INDEL)
IMP	PA0141	SLY877	100

B

Gene	OY-M	AYWB	% similarity (DNA)
tuf	PAM256	AYWB456	97
eno	PAM284	AYWB437	96
rplA	PAM257	AYWB464	96
rpoB	PAM260	AYWB461	96
dnaA	PAM001	AYWB001	94
tmk-b	PAM230	AYWB492	95
secA	PAM474	AYWB304	95 (3 ambiguities)
secE	PAM254	AYWB467	95
secY	PAM220	AYWB502	95
potA	PAM659	AYWB095	96
potB	PAM660	AYWB094	95
potC	PAM661	AYWB093	95
potD	PAM662	AYWB092	91
oppD	PAM192	AYWB528	95
oppE/F	PAM193	AYWB527	94
mgtA	PAM252	AYWB469	94 (over common area)
mgtA	PAM186	AYWB533	94
malE	PAM749	AYWB667	92
IMP	PAM019	AYWB013	94
