# Supplementary material for: Comparison of the complete genome sequence of two closely related isolates of ‘Candidatus Phytoplasma australiense’ reveals genome plasticity
Source: BMC Genomics. 2013 Aug 2;14:529. doi: 10.1186/1471-2164-14-529 (PMC3750655; doi:10.1186/1471-2164-14-529)
Supplement: Additional file 5 — Composition of ‘Candidatus Phytoplasma australiense’ rpoD Groups A and B. SLY570 lacks a 5′ UTR because of the insertion of SLY571, and cannot be placed in GpA or GpB, although the amino acid sequence aligns better with GpA samples than those belonging to GpB. [file 1471-2164-14-529-S5.pdf]

Additional file 5

Table S4. Composition of ‘*Ca*. Phytoplasma australiense’ *rpoD* Group A and Group B. SLY570 lacks a 5’ UTR because of the insertion of SLY571, and cannot be placed in GpA or GpB, although the amino acid sequence aligns better with GpA samples than those belonging to GpB.

	PAa	SLY
GpA	PA0127, PA0258	SLY053, SLY130, SLY160, SLY255, SLY460, SLY641, SLY683, SLY734, SLY787, SLY858, SLY949, SLY980, SLY1020, SLY571, SLY638, SLY1073
GpB	PA0316, PA0409, PA0723, PA0790, PA0815	SLY256, SLY558, SLY1027
Pseudo		SLY570
