# Supplementary material for: Comparison of the complete genome sequence of two closely related isolates of ‘Candidatus Phytoplasma australiense’ reveals genome plasticity
Source: BMC Genomics. 2013 Aug 2;14:529. doi: 10.1186/1471-2164-14-529 (PMC3750655; doi:10.1186/1471-2164-14-529)
Supplement: Additional file 6 — Example of an SLY Open Reading Frame (ORF) being altered by the intrusion of the 5′ Untranslated Region (UTR) of the rpoD gene. (A) Diagram illustrating that the 150 bp 5′ UTR of SLY1027 (rpoD Group B) overlaps with the 3′ region of the ORF SLY1026. (B) A line-up of predicted amino acid sequences of SLY1026 and paralogues shows a high degree of sequence identity except for 11 residues of the carboxyl (3′) terminus. (C) Nucleic acid alignment of SLY1026 and paralogues shows that the point at which sequences differ corresponds to the 5′UTR of SLY1027 (bottom row). Yellow colouring indicates exact nucleotide match and blue colouring is greatest consensus match. [file 1471-2164-14-529-S6.pdf]

Additional file 6

Figure S2. Example of an SLY Open Reading Frame (ORF) being altered by the intrusion of the 5′ Untranslated Region (UTR) of the *rpoD* gene. (A) Diagram illustrating that the 150 bp 5′ UTR of SLY1027 (*rpoD* Group B) overlaps with the 3′ region of the ORF SLY1026. (B) A line-up of predicted amino acid sequences of SLY1026 and paralogues shows a high degree of sequence identity except for 11 residues of the carboxyl (3′) terminus. (C) Nucleic acid alignment of SLY1026 and paralogues shows that the point at which sequences differ corresponds to the 5′UTR of SLY1027 (bottom row). Yellow colouring indicates exact nucleotide match and blue colouring is greatest consensus match.

A

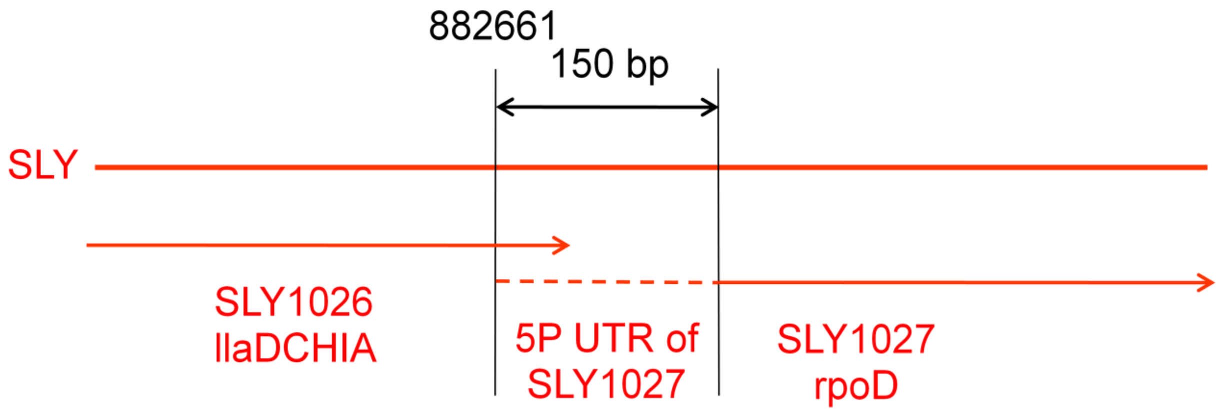

B

```
SLY_1026 (190) WR-EGIKDKDFYQKPFTFEDHKRLYKSLEMANYRKAKWLYTNYNSSKITELFKKFHIQPIQTTTKHNLTSKGWTDEIIRTLFV
SLY_1079 (190) WR-EGIKDKYFYQKPFTFEDHKRLYKSLEMANYRKVKWLYTNYNSSKITELFKKFHIQPIQTTTKHNLTSKGISQEVIIKNY-
 SLY_058 (190) WR-EGIKDKYFYQKPFTFEDHKRLYKSLEMANYRKVKWLYTNYNSSKITELFKKFHIQPIQTTTKHNLTSKGISQEVIIKNY-
 SLY_729 (190) WR-EGIKDKYFYQKPFTFEDHKRLYKSLEMANYRKVKWLYTNYNSSKITELFKKFHIQPIQTTTKHNLTSKGISQEVIIKNY-
SLY_1088 (190) WR-EGIKDKYFYQKPFTFEDHKRLYKSLEMANYRKVKWLYTNYNSSKITELFKKFHIQPIQTTTKYSLTSKGISQEVIIKNY-
 SLY_171 (190) WR-EGIKDKYFYQKPFTFEDHKRLYKSLEMANYRKVKWLYTNYNSSKITELFKKFHIQPIQTTTKHNLTSKGISQEVIIKNY-
 SLY_582 (190) WR-EGIKDKYFYQKPFTFEDHKRLYKSLEMANYRKVKWLYTNYNSSKITELFKKFHIQPIQTTTKHNLTSKGISQEVIIKNY-
 SLY_693 (190) WR-EGIKDKVFYQKPFTFEDHKRLYKSLEMANYRKVKWLYTNYNSSKITELFKKFHIQPIQTTTKHNLTSKGISQEVIIKNY-
 SLY_959 (190) WR-EGIKDKVFYQKPFTFEDHKRLYKSLEMANYRKVKWLYTNYNSSKITELFKKFHIQPIQTTTKHNLTSKGISQEVIIKNY-
 SLY_985 (190) WR-EGIKDKVFYQKPFTFEDHKRLYKSLEMANYRKVKWLYTNYNSSKITELFKKFHIQPIQTTTKHNLTSKGISQEVIIKNY-
 SLY_218 (190) WR-EGTKDKDFYQKPFTFEDHKRLYKSLEMANYRKVKWLYTNYNSPKIIELFKDFHIQPLQTTTNYALTSKGITQEVIIKNY-
  PA0250 (190) WR-EGTKDKDFYQKPFTFEDHKRLYKSLEMANYRKVKWLYTNYNSPKIIELFKDFHIQPLQTTTNYALTSKGITQEVIIKNY-
 SLY_829 (129) WR-EGTKDKDFYQKPFTFEDHKRLYKSLEMANYRKVKWLYTNYNSPKIIELFKDFHIQPIQTTTNYALTSKGITQEVIIKNY-
```

C

```
          (744) 744  750       760       770       780       790       800              819
 SLY_058 (744) TCAAACCACTACAAAACACAATTTAACTTCAAAAGGGATATCGCAAGAAGTTATTATTAAAAATTATTAA------
 SLY_729 (744) TCAAACCACTACAAAACACAATTTAACTTCAAAAGGGATATCGCAAGAAGTTATTATTAAAAATTATTAA------
SLY_1079 (744) TCAAACCACTACAAAACACAATTTAACTTCAAAAGGGATATCGCAAGAAGTTATTATTAAAAATTATTAA------
SLY_1026 (744) TCAAACCACTACAAAACACAATTTAACTTCAAAAGGGTGGACTGATGAAATAATTAGGACACTTTTTGTTTTTTAA
SLY_1088 (744) TCAAACCACTACAAAATACAGTTTAACTTCAAAAGGGATATCACAAGAAGTTATTATTAAAAATTATTAA------
 SLY_171 (744) TCAAACCACTACAAAACACAATTTAACTTCAAAAGGGATATCGCAAGAAGTTATTATTAAAAATTATTAA------
 SLY_582 (744) TCAAACCACTACAAAACACAATTTAACTTCAAAAGGGATATCGCAAGAAGTTATTATTAAAAATTATTAA------
 SLY_693 (744) TCAAACCACTACAAAACACAATTTAACTTCAAAAGGGATATCGCAAGAAGTTATTATTAAAAATTATTAA------
 SLY_985 (744) TCAAACCACTACAAAACACAATTTAACTTCAAAAGGGATATCGCAAGAAGTTATTATTAAAAATTATTAA------
 SLY_959 (744) TCAAACCACTACAAAACACAATTTAACTTCAAAAGGGATATCGCAAGAAGTTATTATTAAAAATTATTAA------
 SLY_218 (744) CCAAACTACAACCAATTACGCTTTAACTTCAAAAGGAATAACACAAGAAGTTATTATTAAAAATTATTAA------
 SLY_829 (561) TCAAACTACAACCAATTACGCTTTAACTTCAAAAGGAATAACACAAGAAGTTATTATTAAAAATTATTAA------
 SLY1027   (1) -------------------------------------TGGACTGATGAAATAATTAGGACACTTTTTGTTTTTTAA
```
