# Supplementary material for: Comparison of the complete genome sequence of two closely related isolates of ‘Candidatus Phytoplasma australiense’ reveals genome plasticity
Source: BMC Genomics. 2013 Aug 2;14:529. doi: 10.1186/1471-2164-14-529 (PMC3750655; doi:10.1186/1471-2164-14-529)
Supplement: Additional file 7 — Junctions associated with 5′ Untranslated Region of rpoD. Junctions associated with 5′ Untranslated Region of rpoD of ‘Candidatus Phytoplasma australiense’ isolates PAa and SLY. [file 1471-2164-14-529-S7.pdf]

Additional file 7

Table S5. Junctions associated with 5′ Untranslated Region of *rpoD* of ‘*Ca*. Phytoplasma australiense’ isolates PAa and SLY.

Junction Description	*rpo*D genes involved
SLY Repeat #1	SLY160 & SLY571
Junction middle box J	SLY460
Junction end Box K	SLY683
Junction start box F1	SLY 734
Junction start box C	SLY 787
Junction end Box L	SLY1020
Junction end Box M	SLY1073
Junction start Box M	PA0815
