# Supplementary material for: Comparison of the complete genome sequence of two closely related isolates of ‘Candidatus Phytoplasma australiense’ reveals genome plasticity
Source: BMC Genomics. 2013 Aug 2;14:529. doi: 10.1186/1471-2164-14-529 (PMC3750655; doi:10.1186/1471-2164-14-529)
Supplement: Additional file 8 — Putative modification methylases in ‘Candidatus Phytoplasma australiense’. Putative modification methylases in ‘Ca. Phytoplasma australiense’ isolates PAa and SLY. [file 1471-2164-14-529-S8.pdf]

Additional file 8

Table S6. Putative modification methylases in ‘*Ca*. Phytoplasma australiense’ isolates PAa and SLY.

		SLY		PAa
	Copies + frags	ORFs - fragments (in brackets)	Copies + frags	ORFs - fragments (in brackets)
llaDCHIA	11+2	SLY058, SLY1026, SLY1079, SLY1088, SLY171, SLY218, SLY582, SLY693, SLY729, (SLY829), (SLY851), SLY959, SLY985	1	PA0250
Putative methylase	1	(SLY507)[1] (amino terminus)	2 + 5	(PA0253), PA0291, (PA0352), (PA0353), (PA0621), PA0789, (PA0810)
Putative N-6-adenine specific DNA methylase	1	SLY507[1] (between amino and caboxy paralogs))	1	PA0654
*hpa*IM	2	SLY507[1] (carboxy terminus), SLY556	2	PA0655, PA0722
$CHP_{methylase}$	13 + 3	SLY061, SLY122, SLY176, (SLY221), (SLY212), (SLY352), SLY587, SLY621, SLY700, SLY726, SLY779, SLY824, SLY932, SLY966, SLY989, SLY1012,	12 + 1	PA0043, PA0069, PA0199, PA0231, PA0320, PA0327, PA0377, (PA0400), PA0421, PA0433, PA0739, PA0778, PA0803
*xor*IIM	5 + 5	(SLY005), (SLY006), (SLY007), SLY163, SLY574, SLY634, SLY687, SLY953, (SLY1040), (SLY1041)	1 (as 2 fragments)	(003), (004)
N-6 DNA methylase		-	1	PA0438
yhhF	1	SLY289	1	PA0503

[1]The predicted protein sequence for SLY507 spans regions of a number of methylases.
