# Supplementary material for: Comparison of the complete genome sequence of two closely related isolates of ‘Candidatus Phytoplasma australiense’ reveals genome plasticity
Source: BMC Genomics. 2013 Aug 2;14:529. doi: 10.1186/1471-2164-14-529 (PMC3750655; doi:10.1186/1471-2164-14-529)
Supplement: Additional file 9 — Conserved sequences associated with ltrA in ‘Candidatus Phytoplasma australiense’. Phytoplasma australiense’. Yellow colouring indicates exact nucleotide match and blue colouring is greatest consensus match. (A) Graphical representation of the 5′ Untranslated Region (UTR) (607 bp), Open Reading Frame (1746 bp) and 3′ UTR (143 bp) of SLY; (B) line-up of the approximately the 5′ most 90 bp of the 5′ UTR showing the boundary of the conserved sequence; (C) alignment of c. 150 bp of the 3′ UTR. Lowest sequence of the alignment represents the 3′ end of the ltrA ORF. [file 1471-2164-14-529-S9.pdf]

Additional file 9

Figure S3. Conserved sequences associated with *ltrA* in '*Ca*. Phytoplasma australiense'. Yellow colouring indicates exact nucleotide match and blue colouring is greatest consensus match. (A) Graphical representation of the 5′ Untranslated Region (UTR) (607 bp), Open Reading Frame (1746 bp) and 3' UTR (143 bp) of SLY; (B) line-up of the approximately the 5′ most 90 bp of the 5′ UTR showing the boundary of the conserved sequence; (C) alignment of c. 150 bp of the 3′ UTR. Lowest sequence of the alignment represents the 3′ end of the *ltrA* ORF.

A

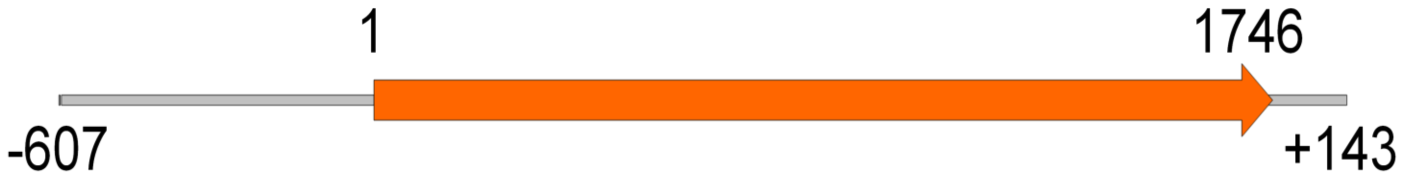

B

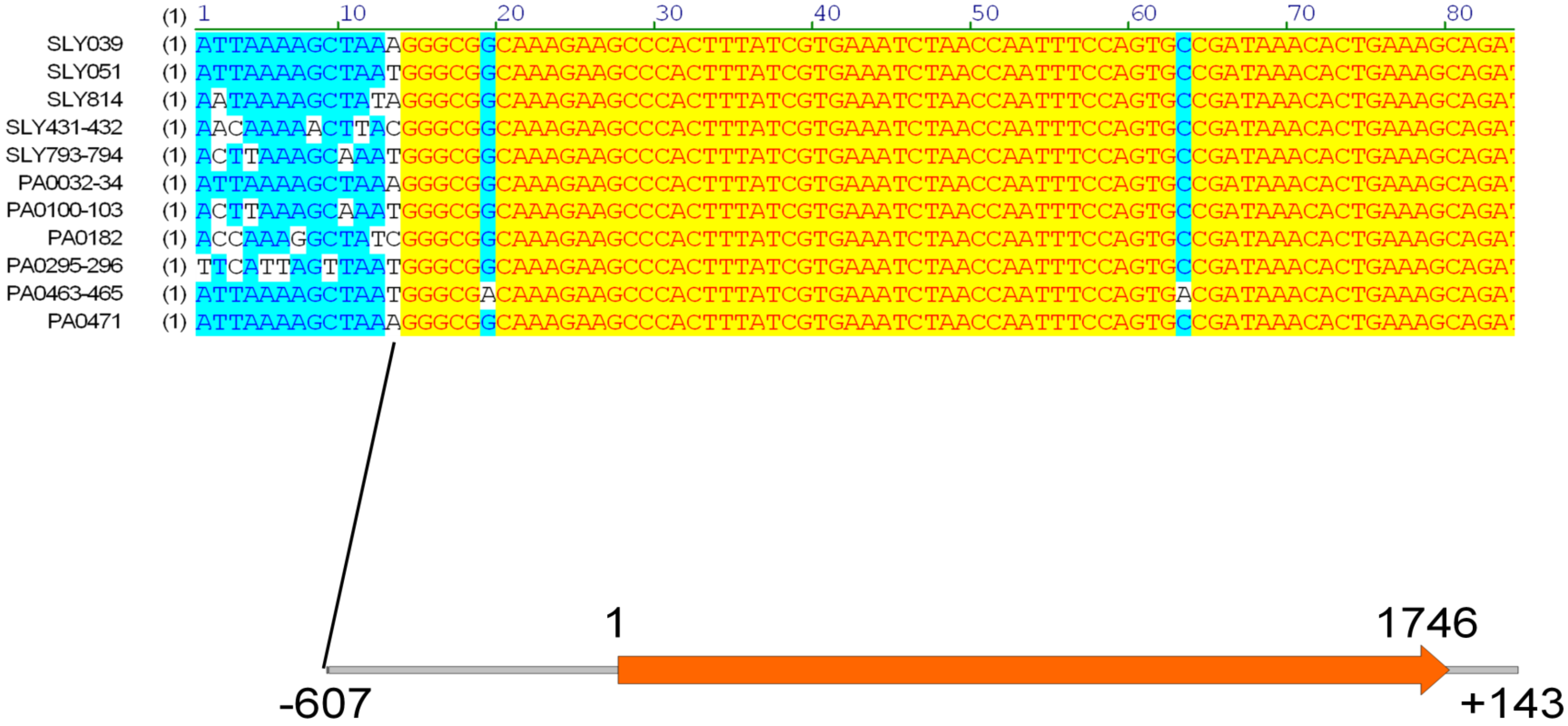

(1) 1 10 20 30 40 50 60 70 80
SLY039 (1) ATTAAAAGCTAAAGGGCGGCAAAGAAGCCCACTTTATCGTGAAATCTAACCAATTTCCAGTGCCGATAAACACTGAAAGCAGA
SLY051 (1) ATTAAAAGCTAATGGGCGGCAAAGAAGCCCACTTTATCGTGAAATCTAACCAATTTCCAGTGCCGATAAACACTGAAAGCAGA
SLY814 (1) AATAAAAGCTATAGGGCGGCAAAGAAGCCCACTTTATCGTGAAATCTAACCAATTTCCAGTGCCGATAAACACTGAAAGCAGA
SLY431-432 (1) AACAAAAACTTACGGGCGGCAAAGAAGCCCACTTTATCGTGAAATCTAACCAATTTCCAGTGCCGATAAACACTGAAAGCAGA
SLY793-794 (1) ACTTAAAGCAAATGGGCGGCAAAGAAGCCCACTTTATCGTGAAATCTAACCAATTTCCAGTGCCGATAAACACTGAAAGCAGA
PA0032-34 (1) ATTAAAAGCTAAAGGGCGGCAAAGAAGCCCACTTTATCGTGAAATCTAACCAATTTCCAGTGCCGATAAACACTGAAAGCAGA
PA0100-103 (1) ACTTAAAGCAAATGGGCGGCAAAGAAGCCCACTTTATCGTGAAATCTAACCAATTTCCAGTGCCGATAAACACTGAAAGCAGA
PA0182 (1) ACCAAAGGCTATCGGGCGGCAAAGAAGCCCACTTTATCGTGAAATCTAACCAATTTCCAGTGCCGATAAACACTGAAAGCAGA
PA0295-296 (1) TTCATTAGTTAATGGGCGGCAAAGAAGCCCACTTTATCGTGAAATCTAACCAATTTCCAGTGCCGATAAACACTGAAAGCAGA
PA0463-465 (1) ATTAAAAGCTAATGGGCGACAAAGAAGCCCACTTTATCGTGAAATCTAACCAATTTCCAGTGACGATAAACACTGAAAGCAGA
PA0471 (1) ATTAAAAGCTAAAGGGCGGCAAAGAAGCCCACTTTATCGTGAAATCTAACCAATTTCCAGTGCCGATAAACACTGAAAGCAGA
1
1746
-607
+143

C

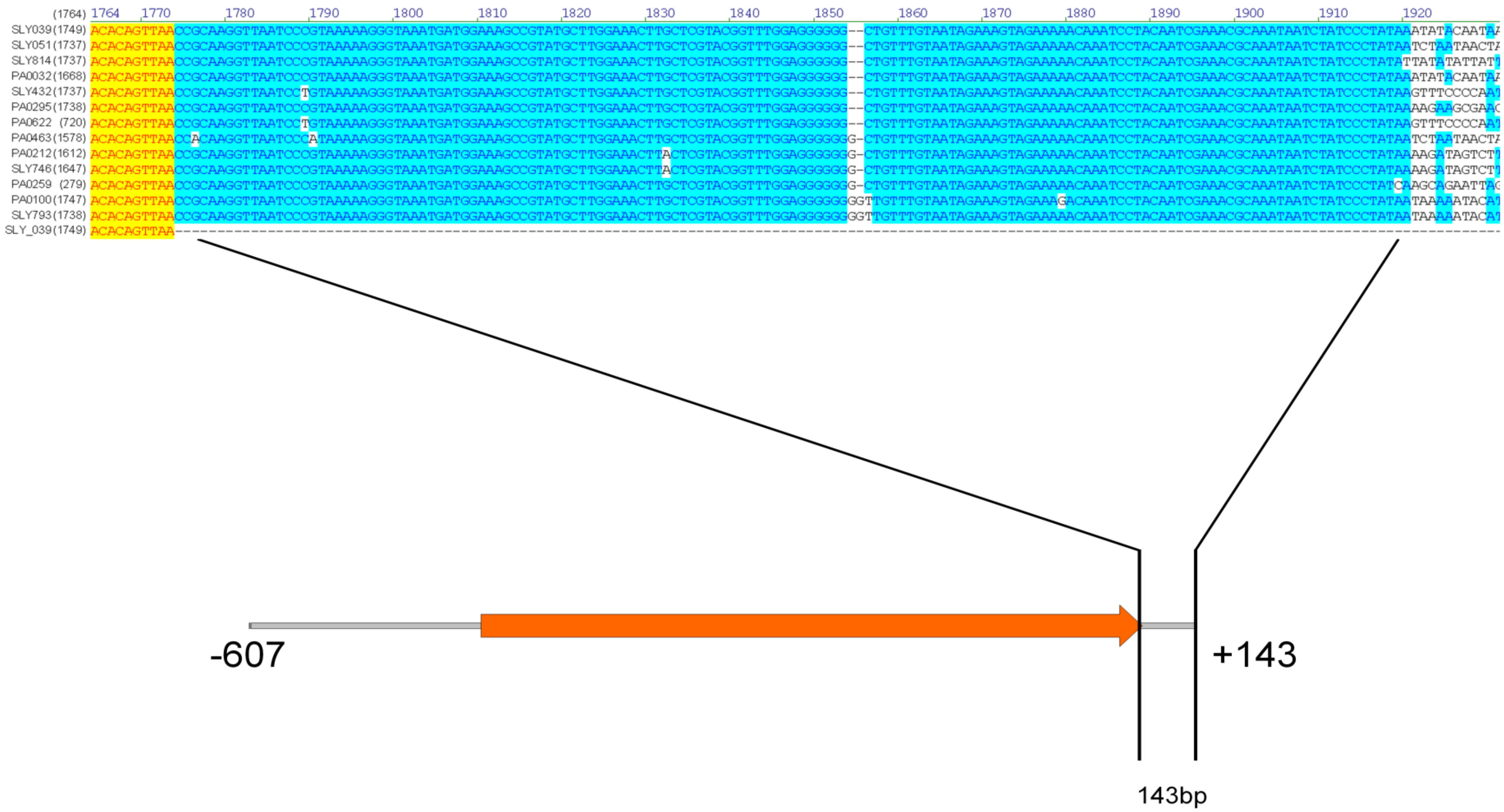
(1764)
1764
1770
1780
1790
1800
1810
1820
1830
1840
1850
1860
1870
1880
1890
1900
1910
1920
SLY039(1749)
SLY051(1737)
SLY814(1737)
PA0032(1668)
SLY432(1737)
PA0295(1738)
PA0622 (720)
PA0463(1578)
PA0212(1612)
SLY746(1647)
PA0259 (279)
PA0100(1747)
SLY793(1738)
SLY_039(1749)
-607
+143
143bp
